# Supplementary material for: Mitochondrial Disruption by Amyloid Beta 42 Identified by Proteomics and Pathway Mapping
Source: Cells. 2021 Sep 10;10(9):2380. doi: 10.3390/cells10092380 (PMC8468661; doi:10.3390/cells10092380)
Supplement: Supplementary file 1 [file cells-10-02380-s001.zip › cells-1332598-supplementary.pdf]

Supplementary Material

Supplement Tables

Supplement Table S1: All proteins significantly decreased by Aβ_{42} treatment, pg.2
Supplement Table S2: All proteins significantly increased by Aβ_{42} treatment, pg. 9
Supplement Table S3: Membrane proteins significantly decreased by Aβ_{42} treatment, pg. 12
Supplement Table S4: Membrane proteins significantly increased by Aβ_{42} treatment, pg. 28
Supplement Table S5: Aβ_{42} proteomic results uploaded to Pathway Studio for GSEA, pg. 19
Supplement Table S6: Mitochondrial proteins significantly decreased by Aβ_{42} treatment, pg. 32
Supplement Table S7: Mitochondrial proteins significantly increased by Aβ_{42} treatment, pg. 36
Supplement Table S8**:** Nuclear proteins significantly decreased by Aβ_{42} treatment, pg. 36
Supplement Table S9: Nuclear proteins significantly increased by Aβ_{42} treatment, pg. 38
Supplement Table S10: Genomic Enrichment Analysis Results, pg. 39
Supplement Table S11: ER proteins significantly decreased by Aβ_{42} treatment, pg. 43
Supplement Table S12: ER proteins significantly decreased by Aβ_{42} treatment, pg. 44

Supplement Table S1: All proteins significantly decreased by 3-day Aβ_{42} treatment (226)

Protein Symbol	Description	Log_2 Fold Change	p-value
LBR	lamin-B receptor	-1.272	2.68E-02
SLC38A2	PREDICTED: sodium-coupled neutral amino acid transporter 2 isoform X1	-1.214	3.85E-02
CBX3	PREDICTED: chromobox protein homolog 3 isoform X1	-6.644	2.37E-16
NDUFA4L2	NADH dehydrogenase [ubiquinone] 1 alpha subcomplex subunit 4-like 2	-1.911	8.12E-09
USMG5	PREDICTED: up-regulated during skeletal muscle growth protein 5	-1.862	5.41E-05
SSR1	PREDICTED: translocon-associated protein subunit alpha isoform X1	-1.258	2.17E-04
SLC7A1	high affinity cationic amino acid transporter 1	-6.644	2.37E-16
TRAPPC10	trafficking protein particle complex subunit 10	-6.644	2.37E-16
EBAG9	PREDICTED: receptor-binding cancer antigen expressed on SiSo cells isoform X1	-6.644	2.37E-16
TCF12	transcription factor 12	-6.644	2.37E-16
LARP4B	la-related protein 4B	-6.644	2.37E-16
WARS	PREDICTED: tryptophan--tRNA ligase, cytoplasmic isoform X1	-1.221	4.51E-02
ALG9	PREDICTED: alpha-1,2-mannosyltransferase ALG9 isoform X1	-1.234	3.51E-02
DNAJC16	dnaJ homolog subfamily C member 16 precursor	-1.329	3.15E-03
SMURF1	PREDICTED: E3 ubiquitin-protein ligase SMURF1 isoform X1	-6.644	2.37E-16
RBM3	PREDICTED: RNA-binding protein 3 isoform X2	-1.523	4.88E-02
VDAC3	PREDICTED: voltage-dependent anion-selective channel protein 3 isoform X1	-1.188	2.96E-02
MTCH2	mitochondrial carrier homolog 2 isoform 1x	-1.474	5.34E-06
CPNE1	copine 1	-6.644	2.37E-16
RGD1562394	PREDICTED: 60S ribosomal protein L30-like	-6.644	2.37E-16
RFC5	replication factor C subunit 5	-1.462	4.96E-03
CTNNA2	PREDICTED: catenin alpha-2 isoform X1	-1.258	2.17E-02
NIPA2	PREDICTED: magnesium transporter NIPA2 isoform X1	-0.905	1.08E-02
PRSS1	anionic trypsin-1 precursor	-1.396	5.70E-06
NDUFV2	NADH dehydrogenase [ubiquinone] flavoprotein 2, mitochondrial precursor	-1.120	4.57E-02
CCHCR1	coiled-coil alpha-helical rod protein 1	-1.006	3.82E-02
PPARD	PREDICTED: peroxisome proliferator-activated receptor delta isoform X1	-2.000	8.57E-06

NOSIP	nitric oxide synthase-interacting protein	-1.358	2.77E-02
TMEM126A	PREDICTED: transmembrane protein 126A isoform X1	-6.644	2.37E-16
VAPA	PREDICTED: vesicle-associated membrane protein-associated protein A isoform X1	-0.727	3.79E-02
SYNJ2BP	synaptojanin-2-binding protein	-1.419	3.59E-03
RGD1305045	PREDICTED: ER membrane protein complex subunit 7 isoform X1	-6.644	2.37E-16
OSTC	oligosaccharyltransferase complex subunit OSTC	-1.279	5.78E-03
SLC25A13	PREDICTED: calcium-binding mitochondrial carrier protein Aralar2 isoform X1	-6.644	2.37E-16
ABCB7	ATP-binding cassette sub-family B member 7, mitochondrial	-1.231	1.72E-02
NDUFC2	NADH dehydrogenase [ubiquinone] 1 subunit C2	-1.336	4.42E-02
VDAC2	PREDICTED: voltage-dependent anion-selective channel protein 2 isoform X1	-0.855	3.40E-02
UQCRC1	cytochrome b-c1 complex subunit 1, mitochondrial precursor	-1.582	3.61E-12
RAP1A	ras-related protein Rap-1A precursor	-6.644	2.37E-16
TYMP	PREDICTED: thymidine phosphorylase isoform X1	-1.737	6.55E-04
COBRA1	negative elongation factor B	-6.644	2.37E-16
DSG3	PREDICTED: desmoglein-3 isoform X1	-1.201	2.51E-02
UQCRH	cytochrome b-c1 complex subunit 6, mitochondrial	-2.506	1.41E-12
PHB2	prohibitin-2	-1.591	1.93E-07
COX6C	cytochrome c oxidase subunit 6C-2	-1.751	5.08E-04
HDHD2	haloacid dehalogenase-like hydrolase domain-containing protein 2 precursor	-1.667	5.92E-04
NDUFA9	NADH dehydrogenase [ubiquinone] 1 alpha subcomplex subunit 9, mitochondrial	-1.868	5.13E-05
LOC102548267	PREDICTED: histocompatibility antigen 60b-like	-1.699	5.25E-04
MYADM	myeloid-associated differentiation marker	-1.130	2.53E-03
TRIAP1	PREDICTED: TP53-regulated inhibitor of apoptosis 1 isoform X1	-1.498	3.47E-06
SEC62	translocation protein SEC62	-0.991	2.98E-02
TOR1AIP1	torsin-1A-interacting protein 1	-1.248	2.05E-02
ZFP819	PREDICTED: zinc finger protein 175 isoform X2	-1.214	3.82E-05
UTP23	rRNA-processing protein UTP23 homolog	-1.059	5.07E-03
NDUFA5	NADH dehydrogenase [ubiquinone] 1 alpha subcomplex subunit 5	-1.020	1.09E-02
DHRS7B	dehydrogenase/reductase SDR family member 7B	-6.644	2.37E-16
NNT	PREDICTED: NAD(P) transhydrogenase, mitochondrial isoform X1	-2.017	4.92E-06
AMY1A	alpha-amylase 1 precursor	-0.819	3.54E-02
NAPA	alpha-soluble NSF attachment protein	-0.806	1.34E-02

PTPLAD1	very-long-chain (3R)-3-hydroxyacyl-CoA dehydratase 3	-1.117	7.22E-04
ATP5H	ATP synthase subunit d, mitochondrial	-1.932	2.37E-16
COX5A	cytochrome c oxidase subunit 5A, mitochondrial precursor	-0.604	3.20E-02
UQCRB	cytochrome b-c1 complex subunit 7	-1.531	1.14E-05
DENND3	PREDICTED: DENN domain-containing protein 3 isoform X1	-6.644	2.37E-16
MEST	mesoderm-specific transcript homolog protein	-0.87	4.08E-02
PDHA1	pyruvate dehydrogenase E1 component subunit alpha, somatic form, mitochondrial precursor	-1.083	1.35E-05
TRIM39	PREDICTED: E3 ubiquitin-protein ligase TRIM39 isoform X1	-0.837	3.54E-02
UQCRFS1	cytochrome b-c1 complex subunit Rieske, mitochondrial	-6.644	2.37E-16
SSR4	translocon-associated protein subunit delta precursor	-0.847	8.35E-03
DDOST	dolichyl-diphosphooligosaccharide--protein glycosyltransferase 48 kDa subunit precursor	-0.793	1.44E-03
SLC25A1	tricarboxylate transport protein, mitochondrial precursor	-2.152	2.37E-16
CBX1	PREDICTED: chromobox protein homolog 1 isoform X1	-6.644	2.37E-16
ATP5C1	ATP synthase subunit gamma, mitochondrial	-2.458	2.37E-16
TMED10	transmembrane emp24 domain-containing protein 10 precursor	-0.651	1.65E-02
LOC680316	PREDICTED: LOW QUALITY PROTEIN: cytochrome P450 11B1, mitochondrial-like isoform X2	-0.991	4.63E-04
APOOL	PREDICTED: MICOS complex subunit MIC27 isoform X1	-1.671	9.74E-07
DCUN1D5	DCN1-like protein 5	-0.873	4.78E-02
TMPO	lamina-associated polypeptide 2, isoform beta	-1.077	5.78E-03
LMNA	prelamin-A/C	-1.155	6.81E-07
ATP6V0A1	PREDICTED: V-type proton ATPase 116 kDa subunit a isoform X4	-0.946	3.65E-04
UQCRQ	cytochrome b-c1 complex subunit 8	-1.791	2.75E-07
ANO6	PREDICTED: anoctamin-6 isoform X1	-0.977	6.25E-04
HAT1	PREDICTED: histone acetyltransferase type B catalytic subunit isoform X1	-6.644	2.37E-16
FAM162A	PREDICTED: protein FAM162A isoform X1	-1.415	1.42E-10
COX4I2	PREDICTED: cytochrome c oxidase subunit 4 isoform 2, mitochondrial isoform X1	-1.45	7.46E-10
TMX2	thioredoxin-related transmembrane protein 2 precursor	-1.062	1.07E-03

GOLGA2	PREDICTED: golgin subfamily A member 2 isoform X1	-0.852	4.55E-02
NDUFB10	NADH dehydrogenase [ubiquinone] 1 beta subcomplex subunit 10	-0.908	2.40E-02
TMEM43	transmembrane protein 43	-1.208	1.35E-03
SLC25A22	PREDICTED: mitochondrial glutamate carrier 1 isoform X1	-6.644	2.37E-16
TAGLN3	transgelin-3	-0.966	1.26E-04
AFG3L2	AFG3-like protein 2	-1.065	3.05E-03
SDHC	succinate dehydrogenase cytochrome b560 subunit, mitochondrial	-1.381	8.09E-03
FUBP3	PREDICTED: far upstream element-binding protein 3 isoform X1	-0.905	2.17E-02
ATP5E	ATP synthase subunit epsilon, mitochondrial	-0.855	4.46E-03
SLC6A2	sodium-dependent noradrenaline transporter	-1.133	2.66E-03
SPCS2	signal peptidase complex subunit 2	-1.657	1.56E-07
CENPJ	PREDICTED: centromere protein J isoform X1	-0.96	3.74E-02
SDHB	succinate dehydrogenase [ubiquinone] iron-sulfur subunit, mitochondrial precursor	-1.47	3.01E-07
TDRKH	PREDICTED: tudor and KH domain-containing protein isoform X1	-0.938	7.93E-03
GET4	Golgi to ER traffic protein 4 homolog	-1.466	1.33E-02
HP	haptoglobin precursor	-6.644	2.37E-16
GHR	PREDICTED: growth hormone receptor isoform X1	-1.47	1.18E-02
ANAPC4	anaphase-promoting complex subunit 4	-6.644	2.37E-16
SDHA	succinate dehydrogenase [ubiquinone] flavoprotein subunit, mitochondrial precursor	-1.333	2.05E-09
ABRACL	costars family protein ABRACL	-1.014	1.79E-04
ATP5J2	ATP synthase subunit f, mitochondrial	-2.077	2.37E-16
NDUFA10	NADH dehydrogenase [ubiquinone] 1 alpha subcomplex subunit 10, mitochondrial precursor	-1.023	1.05E-02
CFL1	cofilin-1	-0.597	3.54E-02
SEC11A	PREDICTED: signal peptidase complex catalytic subunit SEC11A isoform X2	-1.388	2.37E-03
LETM1	LETM1 and EF-hand domain-containing protein 1, mitochondrial precursor	-0.980	7.45E-04
TM9SF3	PREDICTED: transmembrane 9 superfamily member 3 isoform X1	-1.218	1.86E-05
VDAC1	voltage-dependent anion-selective channel protein 1	-1.431	1.80E-09
TRPV2	transient receptor potential cation channel subfamily V member 2	-1.262	1.70E-08
ATP5B	ATP synthase subunit beta, mitochondrial precursor	-2.041	2.37E-16
TOMM70A	mitochondrial import receptor subunit TOM70	-1.155	4.46E-05
MRPL9	39S ribosomal protein L9, mitochondrial	-1.515	3.44E-02
TAP2	antigen peptide transporter 2 precursor	-0.977	1.08E-02

PDHB	pyruvate dehydrogenase E1 component subunit beta, mitochondrial precursor	-1.117	5.06E-06
NDUFA13	NADH dehydrogenase [ubiquinone] 1 alpha subcomplex subunit 13	-3.506	2.37E-16
ATP5D	PREDICTED: ATP synthase subunit delta, mitochondrial isoform X1	-3.047	2.37E-16
EMC2	ER membrane protein complex subunit 2	-1.158	2.10E-03
ATP13A1	manganese-transporting ATPase 13A1	-0.894	4.46E-02
COMTD1	catechol O-methyltransferase domain-containing protein 1	-1.290	2.39E-03
CISD1	CDGSH iron-sulfur domain-containing protein 1	-1.502	7.74E-07
LOC103693780	PREDICTED: 2-oxoglutarate dehydrogenase, mitochondrial isoform X2	-0.642	1.88E-02
ATP5F1	ATP synthase F(0) complex subunit B1, mitochondrial precursor	-2.315	2.37E-16
FIS1	PREDICTED: mitochondrial fission 1 protein isoform X1	-1.105	2.99E-03
TMEM120A	transmembrane protein 120A	-1.127	1.28E-03
WDR12	PREDICTED: ribosome biogenesis protein WDR12 isoform X1	-6.644	2.37E-16
TAP1	antigen peptide transporter 1 precursor	-1.404	1.35E-04
PLP2	proteolipid protein 2	-1.381	3.50E-05
DLAT	dihydrolipoyllysine-residue acetyltransferase component of pyruvate dehydrogenase complex, mitochondrial	-1.184	1.17E-05
RPN1	dolichyl-diphosphooligosaccharide--protein glycosyltransferase subunit 1 precursor	-0.651	1.64E-02
ARF1	PREDICTED: ADP-ribosylation factor 1 isoform X1	-0.737	3.92E-03
PAFAH1B1	PREDICTED: platelet-activating factor acetylhydrolase IB subunit alpha isoform X1	-0.573	4.88E-02
SLC25A4	ADP/ATP translocase 1	-2.826	2.37E-16
LDLRAP1	low density lipoprotein receptor adapter protein 1	-6.644	2.37E-16
CKAP4	PREDICTED: cytoskeleton-associated protein 4 isoform X1	-0.813	9.58E-04
ALB	serum albumin precursor	-1.431	7.83E-11
ATP5L	ATP synthase subunit g, mitochondrial	-2.146	2.37E-16
PHB	prohibitin	-1.966	2.37E-16
UQCRC2	cytochrome b-c1 complex subunit 2, mitochondrial precursor	-1.34	4.94E-06
TM9SF4	PREDICTED: transmembrane 9 superfamily member 4 isoform X1	-0.889	2.73E-02
RPN2	PREDICTED: dolichyl-diphosphooligosaccharide--protein glycosyltransferase subunit 2 isoform X1	-1.370	6.20E-10

TMED7	transmembrane emp24 domain-containing protein 7 precursor	-1.482	4.16E-08
ATP5O	ATP synthase subunit O, mitochondrial precursor	-2.065	2.37E-16
SLC25A11	mitochondrial 2-oxoglutarate/malate carrier protein	-2.300	5.25E-13
SLC25A5	ADP/ATP translocase 2	-2.245	2.37E-16
SLC25A10	mitochondrial dicarboxylate carrier	-1.191	8.09E-06
ATP5I	ATP synthase subunit e, mitochondrial	-1.737	8.81E-12
MPC2	mitochondrial pyruvate carrier 2 isoform 1	-1.751	3.03E-08
ATP5A1	ATP synthase subunit alpha, mitochondrial precursor	-1.857	2.37E-16
RAB1	ras-related protein Rab-1A	-0.630	2.19E-02
SLC25A12	PREDICTED: LOW QUALITY PROTEIN: calcium-binding mitochondrial carrier protein Aralar1 isoform X2	-1.791	7.87E-13
LOC100361457 ; ACTG1	PREDICTED: actin, cytoplasmic 2 isoform X1	-0.648	1.71E-02
RSL1D1L1	ribosomal L1 domain containing 1-like 1	-2.245	1.62E-06
EMC1	PREDICTED: ER membrane protein complex subunit 1 isoform X1	-0.881	3.27E-03
SLC25A3	phosphate carrier protein, mitochondrial isoform 2 precursor	-2.531	2.37E-16
RTRAF	PREDICTED: UPF0568 protein C14orf166 homolog isoform X1	-0.628	4.38E-02
STT3A	PREDICTED: dolichyl-diphosphooligosaccharide--protein glycosyltransferase subunit STT3A isoform X1	-1.026	4.81E-04
CYC1	cytochrome c-1	-2.095	3.98E-12
LOC683498	PREDICTED: actin-like isoform X1	-1.657	4.22E-04
MLEC	malectin precursor	-1.211	1.57E-03
YWHAH	14-3-3 protein eta	-0.685	9.45E-03
SLC25A24	calcium-binding mitochondrial carrier protein SCaMC-1	-1.723	1.98E-11
MRPL17	39S ribosomal protein L17, mitochondrial isoform 1 precursor	-1.635	1.22E-03
ACTC1	actin, alpha cardiac muscle 1	-0.876	2.87E-04
TM9SF2	transmembrane 9 superfamily member 2 precursor	-1.105	1.79E-03
SORD	sorbitol dehydrogenase	-1.077	4.69E-02
ATP8A1	PREDICTED: phospholipid-transporting ATPase IA isoform X2	-6.644	2.37E-16
DNM1L	PREDICTED: dynamin-1-like protein isoform X1	-6.644	2.37E-16
RAB3C	ras-related protein Rab-3C	-6.644	2.37E-16
KRAS	PREDICTED: GTPase KRas isoform X3	-1.544	4.83E-02
DDX4	probable ATP-dependent RNA helicase DDX4	-6.644	2.37E-16
YIF1B	PREDICTED: protein YIF1B isoform X1	-6.644	2.37E-16
KRT19	keratin, type I cytoskeletal 19	-6.644	2.37E-16

SSR3	translocon-associated protein subunit gamma	-6.644	2.37E-16
MBOAT7	lysophospholipid acyltransferase 7	-6.644	2.37E-16
MYL1	PREDICTED: myosin light chain 1/3, skeletal muscle isoform X1	-6.644	2.37E-16
TWF2	twinfilin-2	-6.644	2.37E-16
LOC679739	PREDICTED: NADH dehydrogenase [ubiquinone] iron-sulfur protein 6, mitochondrial	-6.644	2.37E-16
ABCD1	ATP-binding cassette sub-family D member 1	-6.644	2.37E-16
WAPAL	PREDICTED: LOW QUALITY PROTEIN: wings apart-like protein homolog isoform X4	-6.644	2.37E-16
KRT2	PREDICTED: keratin, type II cytoskeletal 2 epidermal isoform X1	-6.644	2.37E-16
MRPL49	39S ribosomal protein L49, mitochondrial	-6.644	2.37E-16
NDUFA7	NADH dehydrogenase [ubiquinone] 1 alpha subcomplex subunit 7	-6.644	2.37E-16
EMC6	PREDICTED: ER membrane protein complex subunit 6 isoform X1	-6.644	2.37E-16
CES1A	carboxylesterase 1-like precursor	-6.644	2.37E-16
TMEM167A	PREDICTED: protein kish-A	-6.644	2.37E-16
COMMD2	COMM domain-containing protein 2	-6.644	2.37E-16
ATP5SL	PREDICTED: ATP synthase subunit s-like protein isoform X1	-6.644	2.37E-16
C1QBP	complement component 1 Q subcomponent-binding protein, mitochondrial precursor	-6.644	2.37E-16
C2CD2	C2 domain-containing protein 2	-6.644	2.37E-16
SLC12A6	solute carrier family 12 member 6	-6.644	2.37E-16
KRT10	PREDICTED: keratin, type I cytoskeletal 10 isoform X1	-1.786	1.47E-03
ZYG11B	PREDICTED: protein zyg-11 homolog B isoform X1	-6.644	2.37E-16
CHCHD4	PREDICTED: mitochondrial intermembrane space import and assembly protein 40	-6.644	2.37E-16
KNTC1	PREDICTED: kinetochore-associated protein 1 isoform X1	-6.644	2.37E-16
ABCB8	ATP-binding cassette sub-family B member 8, mitochondrial precursor	-6.644	2.37E-16
STAMBPL1	AMSH-like protease	-6.644	2.37E-16
LOC100359687	PREDICTED: 39S ribosomal protein L1, mitochondrial isoform X1	-6.644	2.37E-16
MED14	mediator of RNA polymerase II transcription subunit 14	-6.644	2.37E-16
NCBP1	nuclear cap-binding protein subunit 1	-6.644	2.37E-16
ATP9B	PREDICTED: probable phospholipid-transporting ATPase IIB isoform X1	-6.644	2.37E-16
CDC37L1	hsp90 co-chaperone Cdc37-like 1	-6.644	2.37E-16

AKAP1	A-kinase anchor protein 1, mitochondrial	-6.644	2.37E-16
MFSD5	molybdate-anion transporter precursor	-6.644	2.37E-16
MAN1B1	PREDICTED: endoplasmic reticulum mannosyl-oligosaccharide 1,2-alpha-mannosidase isoform X1	-6.644	2.37E-16
NDUFA12	NADH dehydrogenase [ubiquinone] 1 alpha subcomplex subunit 12	-6.644	2.37E-16
CCDC90B	coiled-coil domain-containing protein 90B, mitochondrial precursor	-6.644	2.37E-16
MCU	calcium uniporter protein, mitochondrial precursor	-6.644	2.37E-16
MRPL27	39S ribosomal protein L27, mitochondrial	-6.644	2.37E-16
CLIC4	chloride intracellular channel protein 4	-6.644	2.37E-16
NFATC2IP	NFATC2-interacting protein	-6.644	2.37E-16
UQCR10	ubiquinol-cytochrome c reductase complex 7.2kDa protein	-6.644	2.37E-16
POLA1	DNA polymerase alpha catalytic subunit	-6.644	2.37E-16
TMEM160	transmembrane protein 160 precursor	-6.644	2.37E-16
SLC30A9	zinc transporter 9	-6.644	2.37E-16
LIMA1	PREDICTED: LIM domain and actin-binding protein 1 isoform X1	-6.644	2.37E-16
RGD1307554	uncharacterized protein C19orf47 homolog	-6.644	2.37E-16
PRIM1	DNA primase small subunit isoform 1	-6.644	2.37E-16
MAPKAPK2	PREDICTED: MAP kinase-activated protein kinase 2 isoform X1	-6.644	2.37E-16
ZFR2	PREDICTED: zinc finger RNA-binding protein 2 isoform X1	-6.644	2.37E-16
TIMM29	uncharacterized protein C19orf52 homolog	-6.644	2.37E-16
CDIPT	PREDICTED: CDP-diacylglycerol--inositol 3-phosphatidyltransferase isoform X1	-6.644	2.37E-16

Supplement Table S2: All proteins significantly increased by 3-day Aβ_{42} treatment (90)

**Protein Symbol**	**Description**	**Log_2 Fold Change**	**p-values**
POLDIP3	polymerase delta-interacting protein 3	1.232	4.23E-02
BLOC1S6	biogenesis of lysosome-related organelles complex 1 subunit 6	1.859	9.03E-05
CADPS2	PREDICTED: calcium-dependent secretion activator 2 isoform X1	2.163	1.55E-05
GGACT	PREDICTED: gamma-glutamylaminecyclotransferase isoform X1	1.321	7.72E-03
TROAP	tastin	2.748	2.37E-16
ATR	PREDICTED: serine/threonine-protein kinase ATR isoform X3	0.986	2.50E-02

PKIA	PREDICTED: cAMP-dependent protein kinase inhibitor alpha isoform X1	1.496	2.37E-02
IL18RAP	PREDICTED: interleukin-18 receptor accessory protein	2.357	2.37E-16
LOC102556827	PREDICTED: LOW QUALITY PROTEIN: uncharacterized protein LOC102556827	1.945	2.30E-04
VKORC1L1	PREDICTED: vitamin K epoxide reductase complex subunit 1-like protein 1 isoform X1	1.207	4.36E-03
JPT1	hematological and neurological expressed 1 protein	1.938	1.26E-13
LRP6	PREDICTED: low-density lipoprotein receptor-related protein 6 isoform X1	1.634	1.50E-03
PDXK	pyridoxal kinase	1.416	1.08E-02
LOC679594	PREDICTED: ubiquitin-like	1.209	4.31E-02
CYP2J16	PREDICTED: cytochrome P450 2J2-like	0.846	5.20E-03
MMP10	stromelysin-2 precursor	1.428	1.48E-02
FAT1	PREDICTED: protocadherin Fat 1 isoform X1	0.749	8.44E-03
UBE2J1	PREDICTED: ubiquitin-conjugating enzyme E2 J1 isoform X2	2.241	5.92E-04
CHMP1A	charged multivesicular body protein 1a	2.679	2.37E-16
UFM1	ubiquitin-fold modifier 1 precursor	1.530	2.93E-06
MYL12A	PREDICTED: myosin regulatory light chain RLC-A isoform X1	1.188	2.08E-04
TMSB10	PREDICTED: thymosin beta-10	1.692	8.70E-06
SFRP5	secreted frizzled-related protein 5 precursor	2.369	2.15E-05
NUTF2	nuclear transport factor 2	0.723	4.69E-02
CMC1	PREDICTED: COX assembly mitochondrial protein homolog isoform X1	1.439	8.19E-03
UBQLN1	ubiquilin-1	0.677	4.57E-02
PFN2	profilin-2	0.711	1.92E-02
ERC1	PREDICTED: ELKS/Rab6-interacting/CAST family member 1 isoform X1	2.352	1.22E-11
PIGY	protein preY, mitochondrial precursor	1.825	1.26E-04
AKR1A1	alcohol dehydrogenase [NADP(+)]	0.649	3.79E-02
SLC43A2	PREDICTED: large neutral amino acids transporter small subunit 4 isoform X1	2.518	1.98E-11
MPPED2	PREDICTED: metallophosphoesterase MPPED2 isoform X1	2.004	1.33E-03
PLA2G3	group 3 secretory phospholipase A2 precursor	1.149	5.95E-04
RGD1311595	PREDICTED: uncharacterized protein KIAA2026 homolog isoform X1	1.617	5.08E-04
CRH	corticoliberin precursor	1.202	2.91E-02
MYH14	myosin-14	1.021	7.09E-03
ATP5J	PREDICTED: ATP synthase-coupling factor 6, mitochondrial isoform X1	1.526	2.39E-03
LOC103692831	PREDICTED: 60S ribosomal protein L39	0.645	3.96E-02

HINT1	histidine triad nucleotide-binding protein 1	1.732	1.05E-14
PHLPP2	PH domain leucine-rich repeat-containing protein phosphatase 2	1.108	1.62E-02
TIMM8B	mitochondrial import inner membrane translocase subunit Tim8 B	1.112	7.44E-03
HHATL	protein-cysteine N-palmitoyltransferase HHAT-like protein	2.144	1.32E-11
ALMS1	PREDICTED: Alstrom syndrome protein 1 isoform X4	2.209	1.36E-12
TMEM106A	PREDICTED: transmembrane protein 106A isoform X1	2.717	3.96E-13
ACSS2	PREDICTED: acetyl-coenzyme A synthetase, cytoplasmic isoform X1	0.723	1.26E-02
NPC2	epididymal secretory protein E1 precursor	1.163	3.00E-05
RPL21	60S ribosomal protein L21	1.559	2.29E-03
RPLP1	PREDICTED: 60S acidic ribosomal protein P1 isoform X1	1.045	2.37E-05
ATOX1	copper transport protein ATOX1	1.094	1.78E-04
DPYSL3	dihydropyrimidinase-related protein 3	2.231	2.37E-16
MYH9	myosin-9	1.069	1.40E-05
MTPN	myotrophin	1.062	1.60E-05
FKBP1A	peptidyl-prolyl cis-trans isomerase FKBP1A	2.212	2.37E-16
PSAP	prosaposin isoform B preproprotein	0.801	3.48E-03
GSTP1	glutathione S-transferase P	0.888	1.47E-03
CSTF2	PREDICTED: cleavage stimulation factor subunit 2 isoform X1	0.851	4.25E-02
SCG5	neuroendocrine protein 7B2 precursor	1.259	3.85E-04
NEFL	neurofilament light polypeptide	0.902	1.11E-03
CYP51	lanosterol 14-alpha demethylase	0.631	4.78E-02
DBI	acyl-CoA-binding protein	1.681	8.40E-14
PPIA	PREDICTED: peptidyl-prolyl cis-trans isomerase A	0.662	3.16E-02
PCSK1N	PREDICTED: proSAAS	1.422	1.88E-09
MPP6	PREDICTED: MAGUK p55 subfamily member 6 isoform X1	2.084	1.25E-05
CROT	PREDICTED: peroxisomal carnitine O-octanoyltransferase isoform X1	1.754	3.36E-04
DPY30	protein dpy-30 homolog	1.365	8.27E-07
PARP14	poly [ADP-ribose] polymerase 14	1.63	9.04E-03
PEBP1	phosphatidylethanolamine-binding protein 1	0.811	2.98E-03
MYDGF	PREDICTED: myeloid-derived growth factor isoform X2	1.170	1.49E-05
RPS27A	ubiquitin-40S ribosomal protein S27a	1.141	2.36E-06
BLOC1S2	PREDICTED: biogenesis of lysosome-related organelles complex-1 subunit 2 isoform X2	1.799	4.28E-04
EPB41L3	band 4.1-like protein 3	6.644	2.37E-16

MCTS2	PREDICTED: malignant T-cell-amplified sequence 2	6.644	2.37E-16
PPP2R5B	serine/threonine-protein phosphatase 2A 56 kDa regulatory subunit beta isoform	6.644	2.37E-16
TSC22D1	TSC22 domain family protein 1 isoform 1	6.644	2.37E-16
EPB4.1	PREDICTED: protein 4.1 isoform X3	6.644	2.37E-16
SLC44A2	PREDICTED: choline transporter-like protein 2 isoform X1	6.644	2.37E-16
FRMD4A	FERM domain-containing protein 4A	6.644	2.37E-16
GTF3C4	general transcription factor 3C polypeptide 4	6.644	2.37E-16
ANKRD44	PREDICTED: serine/threonine-protein phosphatase 6 regulatory ankyrin repeat subunit B isoform X1	6.644	2.37E-16
NSRP1	nuclear speckle splicing regulatory protein 1	6.644	2.37E-16
LOC103690059	PREDICTED: LOW QUALITY PROTEIN: maltase-glucoamylase, intestinal-like isoform X1	6.644	2.37E-16
IHH	Indian hedgehog protein precursor	6.644	2.37E-16
PDP1	PREDICTED: pyruvate dehydrogenase [acetyl-transferring]-phosphatase 1, mitochondrial isoform X1	6.644	2.37E-16
ITIH6	PREDICTED: LOW QUALITY PROTEIN: inter-alpha-trypsin inhibitor heavy chain H6-like	6.644	2.37E-16
HPDL	4-hydroxyphenylpyruvate dioxygenase-like protein	6.644	2.37E-16
ADAL	PREDICTED: adenosine deaminase-like protein isoform X1	6.644	2.37E-16
PGLYRP3B	PREDICTED: peptidoglycan recognition protein 3 isoform X3	6.644	2.37E-16
RGD1560289	PREDICTED: uncharacterized protein C3orf20 homolog isoform X4	6.644	2.37E-16
SCLY	PREDICTED: selenocysteine lyase isoform X1	6.644	2.37E-16
MSL3	PREDICTED: male-specific lethal 3 homolog isoform X1	6.644	2.37E-16

Supplement Table S3: Membrane proteins significantly decreased by 3-day Aβ_{42} treatment (163)

**Protein Symbol**	**Description**	**Log$_2$ Fold Change**	**p-value**
LBR	lamin-B receptor	-1.272	2.68E-02
SLC38A2	PREDICTED: sodium-coupled neutral amino acid transporter 2 isoform X1	-1.214	3.85E-02
USMG5	PREDICTED: up-regulated during skeletal muscle growth protein 5	-1.862	5.41E-05
SSR1	PREDICTED: translocon-associated protein subunit alpha isoform X1	-1.258	2.17E-04
SLC7A1	high affinity cationic amino acid transporter 1	-6.644	2.37E-16

EBAG9	PREDICTED: receptor-binding cancer antigen expressed on SiSo cells isoform X1	-6.644	2.37E-16
LARP4B	la-related protein 4B	-6.644	2.37E-16
ALG9	PREDICTED: alpha-1,2-mannosyltransferase ALG9 isoform X1	-1.234	3.51E-02
DNAJC16	dnaJ homolog subfamily C member 16 precursor	-1.329	3.15E-03
SMURF1	PREDICTED: E3 ubiquitin-protein ligase SMURF1 isoform X1	-6.644	2.37E-16
VDAC3	PREDICTED: voltage-dependent anion-selective channel protein 3 isoform X1	-1.188	2.96E-02
MTCH2	mitochondrial carrier homolog 2 isoform 1x	-1.474	5.34E-06
CPNE1	copine 1	-6.644	2.37E-16
CTNNA2	PREDICTED: catenin alpha-2 isoform X1	-1.258	2.17E-02
NIPA2	PREDICTED: magnesium transporter NIPA2 isoform X1	-0.905	1.08E-02
NDUFV2	NADH dehydrogenase [ubiquinone] flavoprotein 2, mitochondrial precursor	-1.120	4.57E-02
TMEM126A	PREDICTED: transmembrane protein 126A isoform X1	-6.644	2.37E-16
VAPA	PREDICTED: vesicle-associated membrane protein-associated protein A isoform X1	-0.727	3.79E-02
SYNJ2BP	synaptojanin-2-binding protein	-1.419	3.59E-03
EMC7	PREDICTED: ER membrane protein complex subunit 7 isoform X1	-6.644	2.37E-16
OSTC	oligosaccharyltransferase complex subunit OSTC	-1.279	5.78E-03
SLC25A13	PREDICTED: calcium-binding mitochondrial carrier protein Aralar2 isoform X1	-6.644	2.37E-16
ABCB7	ATP-binding cassette sub-family B member 7, mitochondrial	-1.231	1.72E-02
NDUFC2	NADH dehydrogenase [ubiquinone] 1 subunit C2	-1.336	4.42E-02
VDAC2	PREDICTED: voltage-dependent anion-selective channel protein 2 isoform X1	-0.855	3.40E-02
UQCRC1	cytochrome b-c1 complex subunit 1, mitochondrial precursor	-1.582	3.61E-12
RAP1A	ras-related protein Rap-1A precursor	-6.644	2.37E-16
DSG3	PREDICTED: desmoglein-3 isoform X1	-1.201	2.51E-02
UQCRH	cytochrome b-c1 complex subunit 6, mitochondrial	-2.506	1.41E-12
PHB2	prohibitin-2	-1.591	1.93E-07
COX6C	cytochrome c oxidase subunit 6C-2	-1.751	5.08E-04
HDHD2	haloacid dehalogenase-like hydrolase domain-containing protein 2 precursor	-1.667	5.92E-04
NDUFA9	NADH dehydrogenase [ubiquinone] 1 alpha subcomplex subunit 9, mitochondrial	-1.868	5.13E-05
LOC102548267	PREDICTED: histocompatibility antigen 60b-like	-1.699	5.25E-04
MYADM	myeloid-associated differentiation marker	-1.130	2.53E-03

SEC62	translocation protein SEC62	-0.991	2.98E-02
TOR1AIP1	torsin-1A-interacting protein 1	-1.248	2.05E-02
NDUFA5	NADH dehydrogenase [ubiquinone] 1 alpha subcomplex subunit 5	-1.020	1.09E-02
DHRS7B	dehydrogenase/reductase SDR family member 7B	-6.644	2.37E-16
NNT	PREDICTED: NAD(P) transhydrogenase, mitochondrial isoform X1	-2.017	4.92E-06
NAPA	alpha-soluble NSF attachment protein	-0.806	1.34E-02
PTPLAD1	very-long-chain (3R)-3-hydroxyacyl-CoA dehydratase 3	-1.117	7.22E-04
ATP5H	ATP synthase subunit d, mitochondrial	-1.932	2.37E-16
COX5A	cytochrome c oxidase subunit 5A, mitochondrial precursor	-0.604	3.20E-02
UQCRB	cytochrome b-c1 complex subunit 7	-1.531	1.14E-05
MEST	mesoderm-specific transcript homolog protein	-0.870	4.08E-02
UQCRFS1	cytochrome b-c1 complex subunit Rieske, mitochondrial	-6.644	2.37E-16
SSR4	translocon-associated protein subunit delta precursor	-0.847	8.35E-03
DDOST	dolichyl-diphosphooligosaccharide--protein glycosyltransferase 48 kDa subunit precursor	-0.793	1.44E-03
SLC25A1	tricarboxylate transport protein, mitochondrial precursor	-2.152	2.37E-16
ATP5C1	ATP synthase subunit gamma, mitochondrial	-2.458	2.37E-16
TMED10	transmembrane emp24 domain-containing protein 10 precursor	-0.651	1.65E-02
APOOL	PREDICTED: MICOS complex subunit MIC27 isoform X1	-1.671	9.74E-07
TMPO	lamina-associated polypeptide 2, isoform beta	-1.077	5.78E-03
LMNA	prelamin-A/C	-1.155	6.81E-07
ATP6V0A1	PREDICTED: V-type proton ATPase 116 kDa subunit a isoform X4	-0.946	3.65E-04
UQCRQ	cytochrome b-c1 complex subunit 8	-1.791	2.75E-07
ANO6	PREDICTED: anoctamin-6 isoform X1	-0.977	6.25E-04
FAM162A	PREDICTED: protein FAM162A isoform X1	-1.415	1.42E-10
COX4I2	PREDICTED: cytochrome c oxidase subunit 4 isoform 2, mitochondrial isoform X1	-1.450	7.46E-10
TMX2	thioredoxin-related transmembrane protein 2 precursor	-1.062	1.07E-03
GOLGA2	PREDICTED: golgin subfamily A member 2 isoform X1	-0.852	4.55E-02
NDUFB10	NADH dehydrogenase [ubiquinone] 1 beta subcomplex subunit 10	-0.908	2.40E-02
TMEM43	transmembrane protein 43	-1.208	1.35E-03

SLC25A22	PREDICTED: mitochondrial glutamate carrier 1 isoform X1	-6.644	2.37E-16
AFG3L2	AFG3-like protein 2	-1.065	3.05E-03
SDHC	succinate dehydrogenase cytochrome b560 subunit, mitochondrial	-1.381	8.09E-03
FUBP3	PREDICTED: far upstream element-binding protein 3 isoform X1	-0.905	2.17E-02
ATP5E	ATP synthase subunit epsilon, mitochondrial	-0.855	4.46E-03
SLC6A2	sodium-dependent noradrenaline transporter	-1.133	2.66E-03
SPCS2	signal peptidase complex subunit 2	-1.657	1.56E-07
CENPJ	PREDICTED: centromere protein J isoform X1	-0.960	3.74E-02
SDHB	succinate dehydrogenase [ubiquinone] iron-sulfur subunit, mitochondrial precursor	-1.470	3.01E-07
TDRKH	PREDICTED: tudor and KH domain-containing protein isoform X1	-0.938	7.93E-03
GHR	PREDICTED: growth hormone receptor isoform X1	-1.470	1.18E-02
SDHA	succinate dehydrogenase [ubiquinone] flavoprotein subunit, mitochondrial precursor	-1.333	2.05E-09
ATP5J2	ATP synthase subunit f, mitochondrial	-2.077	2.37E-16
CFL1	cofilin-1	-0.597	3.54E-02
SEC11A	PREDICTED: signal peptidase complex catalytic subunit SEC11A isoform X2	-1.388	2.37E-03
LETM1	LETM1 and EF-hand domain-containing protein 1, mitochondrial precursor	-0.980	7.45E-04
VDAC1	voltage-dependent anion-selective channel protein 1	-1.431	1.80E-09
TRPV2	transient receptor potential cation channel subfamily V member 2	-1.262	1.70E-08
ATP5B	ATP synthase subunit beta, mitochondrial precursor	-2.041	2.37E-16
TOMM70A	mitochondrial import receptor subunit TOM70	-1.155	4.46E-05
TAP2	antigen peptide transporter 2 precursor	-0.977	1.08E-02
NDUFA13	NADH dehydrogenase [ubiquinone] 1 alpha subcomplex subunit 13	-3.506	2.37E-16
ATP5D	PREDICTED: ATP synthase subunit delta, mitochondrial isoform X1	-3.047	2.37E-16
ATP13A1	manganese-transporting ATPase 13A1	-0.894	4.46E-02
COMTD1	catechol O-methyltransferase domain-containing protein 1	-1.290	2.39E-03
CISD1	CDGSH iron-sulfur domain-containing protein 1	-1.502	7.74E-07
LOC103693780	PREDICTED: 2-oxoglutarate dehydrogenase, mitochondrial isoform X2	-0.642	1.88E-02
ATP5F1	ATP synthase F(0) complex subunit B1, mitochondrial precursor	-2.315	2.37E-16

FIS1	PREDICTED: mitochondrial fission 1 protein isoform X1	-1.105	2.99E-03
TMEM120A	transmembrane protein 120A	-1.127	1.28E-03
TAP1	antigen peptide transporter 1 precursor	-1.404	1.35E-04
PLP2	proteolipid protein 2	-1.381	3.50E-05
RPN1	dolichyl-diphosphooligosaccharide--protein glycosyltransferase subunit 1 precursor	-0.651	1.64E-02
ARF1	PREDICTED: ADP-ribosylation factor 1 isoform X1	-0.737	3.92E-03
PAFAH1B1	PREDICTED: platelet-activating factor acetylhydrolase IB subunit alpha isoform X1	-0.573	4.88E-02
SLC25A4	ADP/ATP translocase 1	-2.826	2.37E-16
LDLRAP1	low density lipoprotein receptor adapter protein 1	-6.644	2.37E-16
ATP5L	ATP synthase subunit g, mitochondrial	-2.146	2.37E-16
PHB	prohibitin	-1.966	2.37E-16
UQCRC2	cytochrome b-c1 complex subunit 2, mitochondrial precursor	-1.340	4.94E-06
RPN2	PREDICTED: dolichyl-diphosphooligosaccharide--protein glycosyltransferase subunit 2 isoform X1	-1.370	6.20E-10
TMED7	transmembrane emp24 domain-containing protein 7 precursor	-1.482	4.16E-08
ATP5O	ATP synthase subunit O, mitochondrial precursor	-2.065	2.37E-16
SLC25A11	mitochondrial 2-oxoglutarate/malate carrier protein	-2.300	5.25E-13
SLC25A5	ADP/ATP translocase 2	-2.245	2.37E-16
SLC25A10	mitochondrial dicarboxylate carrier	-1.191	8.09E-06
ATP5I	ATP synthase subunit e, mitochondrial	-1.737	8.81E-12
MPC2	mitochondrial pyruvate carrier 2 isoform 1	-1.751	3.03E-08
ATP5A1	ATP synthase subunit alpha, mitochondrial precursor	-1.857	2.37E-16
RAB1	ras-related protein Rab-1A	-0.630	2.19E-02
SLC25A12	PREDICTED: LOW QUALITY PROTEIN: calcium-binding mitochondrial carrier protein Aralar1 isoform X2	-1.791	7.87E-13
ACTG1	PREDICTED: actin, cytoplasmic 2 isoform X1	-0.648	1.71E-02
RSL1D1L1	ribosomal L1 domain containing 1-like 1	-2.245	1.62E-06
SLC25A3	phosphate carrier protein, mitochondrial isoform 2 precursor	-2.531	2.37E-16
STT3A	PREDICTED: dolichyl-diphosphooligosaccharide--protein glycosyltransferase subunit STT3A isoform X1	-1.026	4.81E-04
CYC1	cytochrome c-1	-2.095	3.98E-12
MLEC	malectin precursor	-1.211	1.57E-03
YWHAH	14-3-3 protein eta	-0.685	9.45E-03
SLC25A24	calcium-binding mitochondrial carrier protein SCaMC-1	-1.723	1.98E-11

MRPL17	39S ribosomal protein L17, mitochondrial isoform 1 precursor	-1.635	1.22E-03
ACTC1	actin, alpha cardiac muscle 1	-0.876	2.87E-04
TM9SF2	transmembrane 9 superfamily member 2 precursor	-1.105	1.79E-03
SORD	sorbitol dehydrogenase	-1.077	4.69E-02
ATP8A1	PREDICTED: phospholipid-transporting ATPase IA isoform X2	-6.644	2.37E-16
DNM1L	PREDICTED: dynamin-1-like protein isoform X1	-6.644	2.37E-16
RAB3C	ras-related protein Rab-3C	-6.644	2.37E-16
KRAS	PREDICTED: GTPase KRas isoform X3	-1.544	4.83E-02
YIF1B	PREDICTED: protein YIF1B isoform X1	-6.644	2.37E-16
KRT19	keratin, type I cytoskeletal 19	-6.644	2.37E-16
SSR3	translocon-associated protein subunit gamma	-6.644	2.37E-16
MBOAT7	lysophospholipid acyltransferase 7	-6.644	2.37E-16
LOC679739	PREDICTED: NADH dehydrogenase [ubiquinone] iron-sulfur protein 6, mitochondrial	-6.644	2.37E-16
ABCD1	ATP-binding cassette sub-family D member 1	-6.644	2.37E-16
KRT2	PREDICTED: keratin, type II cytoskeletal 2 epidermal isoform X1	-6.644	2.37E-16
NDUFA7	NADH dehydrogenase [ubiquinone] 1 alpha subcomplex subunit 7	-6.644	2.37E-16
EMC6	PREDICTED: ER membrane protein complex subunit 6 isoform X1	-6.644	2.37E-16
TMEM167A	PREDICTED: protein kish-A	-6.644	2.37E-16
C1QBP	complement component 1 Q subcomponent-binding protein, mitochondrial precursor	-6.644	2.37E-16
C2CD2	C2 domain-containing protein 2	-6.644	2.37E-16
SLC12A6	solute carrier family 12 member 6	-6.644	2.37E-16
KRT10	PREDICTED: keratin, type I cytoskeletal 10 isoform X1	-1.786	1.47E-03
KNTC1	PREDICTED: kinetochore-associated protein 1 isoform X1	-6.644	2.37E-16
ABCB8	ATP-binding cassette sub-family B member 8, mitochondrial precursor	-6.644	2.37E-16
STAMBPL1	AMSH-like protease	-6.644	2.37E-16
MED14	mediator of RNA polymerase II transcription subunit 14	-6.644	2.37E-16
ATP9B	PREDICTED: probable phospholipid-transporting ATPase IIB isoform X1	-6.644	2.37E-16
AKAP1	A-kinase anchor protein 1, mitochondrial	-6.644	2.37E-16
MFSD5	molybdate-anion transporter precursor	-6.644	2.37E-16
MAN1B1	PREDICTED: endoplasmic reticulum mannosyl-oligosaccharide 1,2-alpha-mannosidase isoform X1	-6.644	2.37E-16
NDUFA12	NADH dehydrogenase [ubiquinone] 1 alpha subcomplex subunit 12	-6.644	2.37E-16

CCDC90B	coiled-coil domain-containing protein 90B, mitochondrial precursor	-6.644	2.37E-16
MCU	calcium uniporter protein, mitochondrial precursor	-6.644	2.37E-16
CLIC4	chloride intracellular channel protein 4	-6.644	2.37E-16
UQCR10	ubiquinol-cytochrome c reductase complex 7.2kDa protein	-6.644	2.37E-16
TMEM160	transmembrane protein 160 precursor	-6.644	2.37E-16
SLC30A9	zinc transporter 9	-6.644	2.37E-16
PRIM1	DNA primase small subunit isoform 1	-6.644	2.37E-16
TIMM29	uncharacterized protein C19orf52 homolog	-6.644	2.37E-16
CDIPT	PREDICTED: CDP-diacylglycerol--inositol 3-phosphatidyltransferase isoform X1	-6.644	2.37E-16

Supplement Table S4: Membrane proteins significantly increased by 3-day Aβ_{42} treatment (36)

**Protein Symbol**	**Description**	**Log_2 Fold Change**	**p-value**
BLOC1S6	biogenesis of lysosome-related organelles complex 1 subunit 6	1.859	9.03E-05
IL18RAP	PREDICTED: interleukin-18 receptor accessory protein	2.357	2.37E-16
VKORC1L1	PREDICTED: vitamin K epoxide reductase complex subunit 1-like protein 1 isoform X1	1.207	4.36E-03
JPT1	hematological and neurological expressed 1 protein	1.938	1.26E-13
LRP6	PREDICTED: low-density lipoprotein receptor-related protein 6 isoform X1	1.634	1.50E-03
CYP2J16	PREDICTED: cytochrome P450 2J2-like	0.846	5.20E-03
FAT1	PREDICTED: protocadherin Fat 1 isoform X1	0.749	8.44E-03
NUTF2	nuclear transport factor 2	0.723	4.69E-02
UBQLN1	ubiquilin-1	0.677	4.57E-02
ERC1	PREDICTED: ELKS/Rab6-interacting/CAST family member 1 isoform X1	2.352	1.22E-11
PIGY	protein preY, mitochondrial precursor	1.825	1.26E-04
AKR1A1	alcohol dehydrogenase [NADP(+)]	0.649	3.79E-02
SLC43A2	PREDICTED: large neutral amino acids transporter small subunit 4 isoform X1	2.518	1.98E-11
MYH14	myosin-14	1.021	7.09E-03
ATP5J	PREDICTED: ATP synthase-coupling factor 6, mitochondrial isoform X1	1.526	2.39E-03
HINT1	histidine triad nucleotide-binding protein 1	1.732	1.05E-14
PHLPP2	PH domain leucine-rich repeat-containing protein phosphatase 2	1.108	1.62E-02
TIMM8B	mitochondrial import inner membrane translocase subunit Tim8 B	1.112	7.44E-03

HHATL	protein-cysteine N-palmitoyltransferase HHAT-like protein	2.144	1.32E-11
TMEM106A	PREDICTED: transmembrane protein 106A isoform X1	2.717	3.96E-13
RPL21	60S ribosomal protein L21	1.559	2.29E-03
MYH9	myosin-9	1.069	1.40E-05
FKBP1A	peptidyl-prolyl cis-trans isomerase FKBP1A	2.212	2.37E-16
GSTP1	glutathione S-transferase P	0.888	1.47E-03
CYP51	lanosterol 14-alpha demethylase	0.631	4.78E-02
DBI	acyl-CoA-binding protein	1.681	8.40E-14
PPIA	PREDICTED: peptidyl-prolyl cis-trans isomerase A	0.662	3.16E-02
MPP6	PREDICTED: MAGUK p55 subfamily member 6 isoform X1	2.084	1.25E-05
PARP14	poly [ADP-ribose] polymerase 14	1.630	9.04E-03
PEBP1	phosphatidylethanolamine-binding protein 1	0.811	2.98E-03
RPS27A	ubiquitin-40S ribosomal protein S27a	1.141	2.36E-06
BLOC1S2	PREDICTED: biogenesis of lysosome-related organelles complex-1 subunit 2 isoform X2	1.799	4.28E-04
EPB41L3	band 4.1-like protein 3	6.644	2.37E-16
EPB4.1	PREDICTED: protein 4.1 isoform X3	6.644	2.37E-16
SLC44A2	PREDICTED: choline transporter-like protein 2 isoform X1	6.644	2.37E-16
IHH	Indian hedgehog protein precursor	6.644	2.37E-16

Supplement Table S5: Aβ_{42} proteomic results uploaded to Pathway Studio for GSEA

**Protein Symbol**	**Description**	**Entrez ID**	**Abundance Ratio**	**p-value**
EPB41L3	band 4.1-like protein 3	116724	100	2.37E-16
MCTS2	PREDICTED: malignant T-cell-amplified sequence 2	689500	100	2.37E-16
PPP2R5B	serine/threonine-protein phosphatase 2A 56 kDa regulatory subunit beta isoform	309179	100	2.37E-16
TSC22D1	TSC22 domain family protein 1 isoform 1	498545	100	2.37E-16
EPB4.1	PREDICTED: protein 4.1 isoform X3	313052	100	2.37E-16
SLC44A2	PREDICTED: choline transporter-like protein 2 isoform X1	363024	100	2.37E-16
FRMD4A	FERM domain-containing protein 4A	307128	100	2.37E-16
GTF3C4	general transcription factor 3C polypeptide 4	685539	100	2.37E-16
ANKRD44	PREDICTED: serine/threonine-protein phosphatase 6 regulatory ankyrin repeat subunit B isoform X1	301415	100	2.37E-16

NSRP1	nuclear speckle splicing regulatory protein 1	303346	100	2.37E-16
LOC103690059	PREDICTED: LOW QUALITY PROTEIN: maltase-glucoamylase, intestinal-like isoform X1	103690 059	100	2.37E-16
IHH	Indian hedgehog protein precursor	84399	100	2.37E-16
PDP1	PREDICTED: pyruvate dehydrogenase [acetyl-transferring]-phosphatase 1, mitochondrial isoform X1	54705	100	2.37E-16
ITIH6	PREDICTED: LOW QUALITY PROTEIN: inter-alpha-trypsin inhibitor heavy chain H6-like	100912 775	100	2.37E-16
HPDL	4-hydroxyphenylpyruvate dioxygenase-like protein	313521	100	2.37E-16
ADAL	PREDICTED: adenosine deaminase-like protein isoform X1	311352	100	2.37E-16
PGLYRP3B	PREDICTED: peptidoglycan recognition protein 3 isoform X3	295180	100	2.37E-16
RGD1560289	PREDICTED: uncharacterized protein C3orf20 homolog isoform X4	500258	100	2.37E-16
SCLY	PREDICTED: selenocysteine lyase isoform X1	363285	100	2.37E-16
MSL3	PREDICTED: male-specific lethal 3 homolog isoform X1	317464	100	2.37E-16
TROAP	tastin	300219	6.717	2.37E-16
TMEM106A	PREDICTED: transmembrane protein 106A isoform X1	287722	6.574	3.96E-13
CHMP1A	charged multivesicular body protein 1a	365024	6.402	2.37E-16
SLC43A2	PREDICTED: large neutral amino acids transporter small subunit 4 isoform X1	287532	5.728	1.98E-11
SFRP5	secreted frizzled-related protein 5 precursor	309377	5.165	2.15E-05
IL18RAP	PREDICTED: interleukin-18 receptor accessory protein	373540	5.122	2.37E-16
ERC1	PREDICTED: ELKS/Rab6-interacting/CAST family member 1 isoform X1	266806	5.104	1.22E-11
UBE2J1	PREDICTED: ubiquitin-conjugating enzyme E2 J1 isoform X2	297961	4.728	5.92E-04
DPYSL3	dihydropyrimidinase-related protein 3	25418	4.695	2.37E-16
FKBP1A	peptidyl-prolyl cis-trans isomerase FKBP1A	25639	4.632	2.37E-16

ALMS1	PREDICTED: Alstrom syndrome protein 1 isoform X4	297408	4.624	1.36E-12
CADPS2	PREDICTED: calcium-dependent secretion activator 2 isoform X1	312166	4.48	1.55E-05
HHATL	protein-cysteine N-palmitoyltransferase HHAT-like protein	301073	4.421	1.32E-11
MPP6	PREDICTED: MAGUK p55 subfamily member 6 isoform X1	362359	4.24	1.25E-05
MPPED2	PREDICTED: metallophosphoesterase MPPED2 isoform X1	362185	4.011	1.33E-03
LOC102556827	PREDICTED: LOW QUALITY PROTEIN: uncharacterized protein LOC102556827	102556827	3.851	2.30E-04
JPT1	hematological and neurological expressed 1 protein	287828	3.831	1.26E-13
BLOC1S6	biogenesis of lysosome-related organelles complex 1 subunit 6	317630	3.628	9.03E-05
PIGY	protein preY, mitochondrial precursor	502782	3.542	1.26E-04
BLOC1S2	PREDICTED: biogenesis of lysosome-related organelles complex-1 subunit 2 isoform X2	293938	3.479	4.28E-04
CROT	PREDICTED: peroxisomal carnitine O-octanoyltransferase isoform X1	83842	3.374	3.36E-04
HINT1	histidine triad nucleotide-binding protein 1	690660	3.321	1.05E-14
TMSB10	PREDICTED: thymosin beta-10	100364435	3.231	8.70E-06
DBI	acyl-CoA-binding protein	25045	3.206	8.40E-14
LRP6	PREDICTED: low-density lipoprotein receptor-related protein 6 isoform X1	312781	3.103	1.50E-03
PARP14	poly [ADP-ribose] polymerase 14	303903	3.095	9.04E-03
RGD1311595	PREDICTED: uncharacterized protein KIAA2026 homolog isoform X1	309307	3.067	5.08E-04
RPL21	60S ribosomal protein L21	79449	2.946	2.29E-03
UFM1	ubiquitin-fold modifier 1 precursor	365797	2.887	2.93E-06
ATP5J	PREDICTED: ATP synthase-coupling factor 6, mitochondrial isoform X1	94271	2.879	2.39E-03
PKIA	PREDICTED: cAMP-dependent protein kinase inhibitor alpha isoform X1	114906	2.82	2.37E-02
CMC1	PREDICTED: COX assembly mitochondrial protein homolog isoform X1	363162	2.712	8.19E-03
MMP10	stromelysin-2 precursor	117061	2.691	1.48E-02

PCSK1N	PREDICTED: proSAAS	108348172	2.679	1.88E-09
PDXK	pyridoxal kinase	83578	2.669	1.08E-02
DPY30	protein dpy-30 homolog	286897	2.575	8.27E-07
GGACT	PREDICTED: gamma-glutamylaminecyclotransferase isoform X1	290500	2.498	7.72E-03
SCG5	neuroendocrine protein 7B2 precursor	25719	2.394	3.85E-04
POLDIP3	polymerase delta-interacting protein 3	315170	2.349	4.23E-02
LOC679594	PREDICTED: ubiquitin-like	679594	2.311	4.31E-02
VKORC1L1	PREDICTED: vitamin K epoxide reductase complex subunit 1-like protein 1 isoform X1	103693015	2.308	4.36E-03
CRH	corticoliberin precursor	81648	2.301	2.91E-02
MYL12A	PREDICTED: myosin regulatory light chain RLC-A isoform X1	501203	2.279	2.08E-04
MYDGF	PREDICTED: myeloid-derived growth factor isoform X2	501282	2.250	1.49E-05
NPC2	epididymal secretory protein E1 precursor	286898	2.239	3.00E-05
PLA2G3	group 3 secretory phospholipase A2 precursor	289733	2.217	5.95E-04
RPS27A	ubiquitin-40S ribosomal protein S27a	81777	2.205	2.36E-06
TIMM8B	mitochondrial import inner membrane translocase subunit Tim8 B	64372	2.162	7.44E-03
PHLPP2	PH domain leucine-rich repeat-containing protein phosphatase 2	498949	2.155	1.62E-02
ATOX1	copper transport protein ATOX1	84355	2.135	1.78E-04
MYH9	myosin-9	25745	2.098	1.40E-05
MTPN	myotrophin	79215	2.088	1.60E-05
RPLP1	PREDICTED: 60S acidic ribosomal protein P1 isoform X1	100360522	2.064	2.37E-05
MYH14	myosin-14	308572	2.030	7.09E-03
ATR	PREDICTED: serine/threonine-protein kinase ATR isoform X3	685055	1.980	2.50E-02
NEFL	neurofilament light polypeptide	83613	1.869	1.11E-03
GSTP1	glutathione S-transferase P	24426	1.851	1.47E-03
CSTF2	PREDICTED: cleavage stimulation factor subunit 2 isoform X1	683927	1.804	4.25E-02
CYP2J16	PREDICTED: cytochrome P450 2J2-like	502969	1.798	5.20E-03
PEBP1	phosphatidylethanolamine-binding protein 1	29542	1.754	2.98E-03
PSAP	prosaposin isoform B preproprotein	25524	1.742	3.48E-03

FAT1	PREDICTED: protocadherin Fat 1 isoform X1	83720	1.681	8.44E-03
NUTF2	nuclear transport factor 2	291981	1.651	4.69E-02
ACSS2	PREDICTED: acetyl-coenzyme A synthetase, cytoplasmic isoform X1	311569	1.651	1.26E-02
PFN2	profilin-2	81531	1.637	1.92E-02
UBQLN1	ubiquilin-1	114590	1.599	4.57E-02
PPIA	PREDICTED: peptidyl-prolyl cis-trans isomerase A	100360977	1.582	3.16E-02
AKR1A1	alcohol dehydrogenase [NADP(+)]	78959	1.568	3.79E-02
LOC103692831	PREDICTED: 60S ribosomal protein L39	103692831	1.564	3.96E-02
CYP51	lanosterol 14-alpha demethylase	25427	1.549	4.78E-02
PAFAH1B1	PREDICTED: platelet-activating factor acetylhydrolase IB subunit alpha isoform X1	83572	0.672	4.88E-02
CFL1	cofilin-1	29271	0.661	3.54E-02
COX5A	cytochrome c oxidase subunit 5A, mitochondrial precursor	252934	0.658	3.20E-02
RTRAF	PREDICTED: UPF0568 protein C14orf166 homolog isoform X1	302247	0.647	4.38E-02
RAB1	ras-related protein Rab-1A	81754	0.646	2.19E-02
LOC103693780	PREDICTED: 2-oxoglutarate dehydrogenase, mitochondrial isoform X2	103693780	0.641	1.88E-02
ACTG1	PREDICTED: actin, cytoplasmic 2 isoform X1	287876	0.638	1.71E-02
TMED10	transmembrane emp24 domain-containing protein 10 precursor	84599	0.637	1.65E-02
RPN1	dolichyl-diphosphooligosaccharide--protein glycosyltransferase subunit 1 precursor	25596	0.637	1.64E-02
YWHAH	14-3-3 protein eta	25576	0.622	9.45E-03
VAPA	PREDICTED: vesicle-associated membrane protein-associated protein A isoform X1	58857	0.604	3.79E-02
ARF1	PREDICTED: ADP-ribosylation factor 1 isoform X1	64310	0.600	3.92E-03
DDOST	dolichyl-diphosphooligosaccharide--protein glycosyltransferase 48 kDa subunit precursor	313648	0.577	1.44E-03
NAPA	alpha-soluble NSF attachment protein	140673	0.572	1.34E-02
CKAP4	PREDICTED: cytoskeleton-associated protein 4 isoform X1	362859	0.569	9.58E-04
AMY1A	alpha-amylase 1 precursor	24203	0.567	3.54E-02

TRIM39	PREDICTED: E3 ubiquitin-protein ligase TRIM39 isoform X1	309591	0.560	3.54E-02
SSR4	translocon-associated protein subunit delta precursor	29435	0.556	8.35E-03
GOLGA2	PREDICTED: golgin subfamily A member 2 isoform X1	64528	0.554	4.55E-02
VDAC2	PREDICTED: voltage-dependent anion-selective channel protein 2 isoform X1	83531	0.553	3.40E-02
ATP5E	ATP synthase subunit epsilon, mitochondrial	245958	0.553	4.46E-03
MEST	mesoderm-specific transcript homolog protein	58827	0.547	4.08E-02
DCUN1D5	DCN1-like protein 5	315405	0.546	4.78E-02
ACTC1	actin, alpha cardiac muscle 1	29275	0.545	2.87E-04
EMC1	PREDICTED: ER membrane protein complex subunit 1 isoform X1	362643	0.543	3.27E-03
TM9SF4	PREDICTED: transmembrane 9 superfamily member 4 isoform X1	296279	0.540	2.73E-02
ATP13A1	manganese-transporting ATPase 13A1	290673	0.538	4.46E-02
NIPA2	PREDICTED: magnesium transporter NIPA2 isoform X1	308667	0.534	1.08E-02
FUBP3	PREDICTED: far upstream element-binding protein 3 isoform X1	362106	0.534	2.17E-02
NDUFB10	NADH dehydrogenase [ubiquinone] 1 beta subcomplex subunit 10	681418	0.533	2.40E-02
TDRKH	PREDICTED: tudor and KH domain-containing protein isoform X1	310652	0.522	7.93E-03
ATP6V0A1	PREDICTED: V-type proton ATPase 116 kDa subunit a isoform X4	29757	0.519	3.65E-04
CENPJ	PREDICTED: centromere protein J isoform X1	305909	0.514	3.74E-02
TAGLN3	transgelin-3	103693564	0.512	1.26E-04
ANO6	PREDICTED: anoctamin-6 isoform X1	315272	0.508	6.25E-04
TAP2	antigen peptide transporter 2 precursor	103689996	0.508	1.08E-02
LETM1	LETM1 and EF-hand domain-containing protein 1, mitochondrial precursor	305457	0.507	7.45E-04
SEC62	translocation protein SEC62	294912	0.503	2.98E-02
LOC680316	PREDICTED: LOW QUALITY PROTEIN: cytochrome P450 11B1, mitochondrial-like isoform X2	680316	0.503	4.63E-04
CCHCR1	coiled-coil alpha-helical rod protein 1	406196	0.498	3.82E-02

ABRACL	costars family protein ABRACL	685045	0.495	1.79E-04
NDUFA5	NADH dehydrogenase [ubiquinone] 1 alpha subcomplex subunit 5	25488	0.493	1.09E-02
NDUFA10	NADH dehydrogenase [ubiquinone] 1 alpha subcomplex subunit 10, mitochondrial precursor	316632	0.492	1.05E-02
STT3A	PREDICTED: dolichyl-diphosphooligosaccharide--protein glycosyltransferase subunit STT3A isoform X1	500972	0.491	4.81E-04
UTP23	rRNA-processing protein UTP23 homolog	299900	0.480	5.07E-03
TMX2	thioredoxin-related transmembrane protein 2 precursor	295701	0.479	1.07E-03
AFG3L2	AFG3-like protein 2	307350	0.478	3.05E-03
TMPO	lamina-associated polypeptide 2, isoform beta	25359	0.474	5.78E-03
SORD	sorbitol dehydrogenase	24788	0.474	4.69E-02
PDHA1	pyruvate dehydrogenase E1 component subunit alpha, somatic form, mitochondrial precursor	29554	0.472	1.35E-05
FIS1	PREDICTED: mitochondrial fission 1 protein isoform X1	288584	0.465	2.99E-03
TM9SF2	transmembrane 9 superfamily member 2 precursor	306197	0.465	1.79E-03
PTPLAD1	very-long-chain (3R)-3-hydroxyacyl-CoA dehydratase 3	300783	0.461	7.22E-04
PDHB	pyruvate dehydrogenase E1 component subunit beta, mitochondrial precursor	289950	0.461	5.06E-06
NDUFV2	NADH dehydrogenase [ubiquinone] flavoprotein 2, mitochondrial precursor	81728	0.460	4.57E-02
TMEM120A	transmembrane protein 120A	288591	0.458	1.28E-03
MYADM	myeloid-associated differentiation marker	369016	0.457	2.53E-03
SLC6A2	sodium-dependent noradrenaline transporter	83511	0.456	2.66E-03
LMNA	prelamin-A/C	60374	0.449	6.81E-07
TOMM70A	mitochondrial import receptor subunit TOM70	304017	0.449	4.46E-05
EMC2	ER membrane protein complex subunit 2	362905	0.448	2.10E-03
DLAT	dihydrolipoyllysine-residue acetyltransferase component of	81654	0.440	1.17E-05

	pyruvate dehydrogenase complex, mitochondrial			
VDAC3	PREDICTED: voltage-dependent anion-selective channel protein 3 isoform X1	83532	0.439	2.96E-02
SLC25A10	mitochondrial dicarboxylate carrier	170943	0.438	8.09E-06
DSG3	PREDICTED: desmoglein-3 isoform X1	291752	0.435	2.51E-02
TMEM43	transmembrane protein 43	362401	0.433	1.35E-03
MLEC	malectin precursor	304543	0.432	1.57E-03
SLC38A2	PREDICTED: sodium-coupled neutral amino acid transporter 2 isoform X1	29642	0.431	3.85E-02
ZFP819	PREDICTED: zinc finger protein 175 isoform X2	308561	0.431	3.82E-05
TM9SF3	PREDICTED: transmembrane 9 superfamily member 3 isoform X1	309475	0.430	1.86E-05
WARS	PREDICTED: tryptophan--tRNA ligase, cytoplasmic isoform X1	314442	0.429	4.51E-02
ABCB7	ATP-binding cassette sub-family B member 7, mitochondrial	302395	0.426	1.72E-02
ALG9	PREDICTED: alpha-1,2-mannosyltransferase ALG9 isoform X1	367083	0.425	3.51E-02
TOR1AIP1	torsin-1A-interacting protein 1	246314	0.421	2.05E-02
SSR1	PREDICTED: translocon-associated protein subunit alpha isoform X1	361233	0.418	2.17E-04
CTNNA2	PREDICTED: catenin alpha-2 isoform X1	297357	0.418	2.17E-02
TRPV2	transient receptor potential cation channel subfamily V member 2	29465	0.417	1.70E-08
LBR	lamin-B receptor	89789	0.414	2.68E-02
OSTC	oligosaccharyltransferase complex subunit OSTC	362040	0.412	5.78E-03
COMTD1	catechol O-methyltransferase domain-containing protein 1	305685	0.409	2.39E-03
DNAJC16	dnaJ homolog subfamily C member 16 precursor	362652	0.398	3.15E-03
SDHA	succinate dehydrogenase [ubiquinone] flavoprotein subunit, mitochondrial precursor	157074	0.397	2.05E-09
NDUFC2	NADH dehydrogenase [ubiquinone] 1 subunit C2	293130	0.396	4.42E-02
UQCRC2	cytochrome b-c1 complex subunit 2, mitochondrial precursor	293448	0.395	4.94E-06
NOSIP	nitric oxide synthase-interacting protein	292894	0.390	2.77E-02

RPN2	PREDICTED: dolichyl-diphosphooligosaccharide--protein glycosyltransferase subunit 2 isoform X1	64701	0.387	6.20E-10
SDHC	succinate dehydrogenase cytochrome b560 subunit, mitochondrial	289217	0.384	8.09E-03
PLP2	proteolipid protein 2	302562	0.384	3.50E-05
SEC11A	PREDICTED: signal peptidase complex catalytic subunit SEC11A isoform X2	65166	0.382	2.37E-03
PRSS1	anionic trypsin-1 precursor	24691	0.380	5.70E-06
TAP1	antigen peptide transporter 1 precursor	24811	0.378	1.35E-04
FAM162A	PREDICTED: protein FAM162A isoform X1	360721	0.375	1.42E-10
SYNJ2BP	synaptojanin-2-binding protein	64531	0.374	3.59E-03
VDAC1	voltage-dependent anion-selective channel protein 1	83529	0.371	1.80E-09
ALB	serum albumin precursor	24186	0.371	7.83E-11
COX4I2	PREDICTED: cytochrome c oxidase subunit 4 isoform 2, mitochondrial isoform X1	84683	0.366	7.46E-10
RFC5	replication factor C subunit 5	304528	0.363	4.96E-03
GET4	Golgi to ER traffic protein 4 homolog	288518	0.362	1.33E-02
SDHB	succinate dehydrogenase [ubiquinone] iron-sulfur subunit, mitochondrial precursor	298596	0.361	3.01E-07
GHR	PREDICTED: growth hormone receptor isoform X1	25235	0.361	1.18E-02
MTCH2	mitochondrial carrier homolog 2 isoform 1x	295922	0.360	5.34E-06
TMED7	transmembrane emp24 domain-containing protein 7 precursor	252889	0.358	4.16E-08
TRIAP1	PREDICTED: TP53-regulated inhibitor of apoptosis 1 isoform X1	108348066	0.354	3.47E-06
CISD1	CDGSH iron-sulfur domain-containing protein 1	294362	0.353	7.74E-07
MRPL9	39S ribosomal protein L9, mitochondrial	310653	0.350	3.44E-02
RBM3	PREDICTED: RNA-binding protein 3 isoform X2	114488	0.348	4.88E-02
UQCRB	cytochrome b-c1 complex subunit 7	362897	0.346	1.14E-05
KRAS	PREDICTED: GTPase KRas isoform X3	24525	0.343	4.83E-02
UQCRC1	cytochrome b-c1 complex subunit 1, mitochondrial precursor	301011	0.334	3.61E-12
PHB2	prohibitin-2	114766	0.332	1.93E-07

MRPL17	39S ribosomal protein L17, mitochondrial isoform 1 precursor	171061	0.322	1.22E-03
SPCS2	signal peptidase complex subunit 2	293142	0.317	1.56E-07
LOC683498	PREDICTED: actin-like isoform X1	102552318	0.317	4.22E-04
HDHD2	haloacid dehalogenase-like hydrolase domain-containing protein 2 precursor	361351	0.315	5.92E-04
APOOL	PREDICTED: MICOS complex subunit MIC27 isoform X1	317191	0.314	9.74E-07
LOC102548267	PREDICTED: histocompatibility antigen 60b-like	102547056	0.308	5.25E-04
SLC25A24	calcium-binding mitochondrial carrier protein SCaMC-1	310791	0.303	1.98E-11
TYMP	PREDICTED: thymidine phosphorylase isoform X1	315219	0.300	6.55E-04
ATP5I	ATP synthase subunit e, mitochondrial	140608	0.300	8.81E-12
COX6C	cytochrome c oxidase subunit 6C-2	54322	0.297	5.08E-04
MPC2	mitochondrial pyruvate carrier 2 isoform 1	100359982	0.297	3.03E-08
KRT10	PREDICTED: keratin, type I cytoskeletal 10 isoform X1	450225	0.290	1.47E-03
UQCRQ	cytochrome b-c1 complex subunit 8	497902	0.289	2.75E-07
SLC25A12	PREDICTED: LOW QUALITY PROTEIN: calcium-binding mitochondrial carrier protein Aralar1 isoform X2	362145	0.289	7.87E-13
ATP5A1	ATP synthase subunit alpha, mitochondrial precursor	65262	0.276	2.37E-16
USMG5	PREDICTED: up-regulated during skeletal muscle growth protein 5	103693430	0.275	5.41E-05
NDUFA9	NADH dehydrogenase [ubiquinone] 1 alpha subcomplex subunit 9, mitochondrial	362440	0.274	5.13E-05
NDUFA4L2	NADH dehydrogenase [ubiquinone] 1 alpha subcomplex subunit 4-like 2	100362331	0.266	8.12E-09
ATP5H	ATP synthase subunit d, mitochondrial	641434	0.262	2.37E-16
PHB	prohibitin	25344	0.256	2.37E-16
PPARD	PREDICTED: peroxisome proliferator-activated receptor delta isoform X1	25682	0.250	8.57E-06
NNT	PREDICTED: NAD(P) transhydrogenase, mitochondrial isoform X1	310378	0.247	4.92E-06
ATP5B	ATP synthase subunit beta, mitochondrial precursor	171374	0.243	2.37E-16

ATP5O	ATP synthase subunit O, mitochondrial precursor	192241	0.239	2.37E-16
ATP5J2	ATP synthase subunit f, mitochondrial	690441	0.237	2.37E-16
CYC1	cytochrome c-1	300047	0.234	3.98E-12
ATP5L	ATP synthase subunit g, mitochondrial	300677	0.226	2.37E-16
SLC25A1	tricarboxylate transport protein, mitochondrial precursor	29743	0.225	2.37E-16
SLC25A5	ADP/ATP translocase 2	25176	0.211	2.37E-16
RSL1D1L1	ribosomal L1 domain containing 1-like 1	108348298	0.211	1.62E-06
SLC25A11	mitochondrial 2-oxoglutarate/malate carrier protein	64201	0.203	5.25E-13
ATP5F1	ATP synthase F(0) complex subunit B1, mitochondrial precursor	100911417	0.201	2.37E-16
ATP5C1	ATP synthase subunit gamma, mitochondrial	116550	0.182	2.37E-16
UQCRH	cytochrome b-c1 complex subunit 6, mitochondrial	366448	0.176	1.41E-12
SLC25A3	phosphate carrier protein, mitochondrial isoform 2 precursor	245959	0.173	2.37E-16
SLC25A4	ADP/ATP translocase 1	85333	0.141	2.37E-16
ATP5D	PREDICTED: ATP synthase subunit delta, mitochondrial isoform X1	245965	0.121	2.37E-16
NDUFA13	NADH dehydrogenase [ubiquinone] 1 alpha subcomplex subunit 13	100911483	0.088	2.37E-16
CBX3	PREDICTED: chromobox protein homolog 3 isoform X1	297093	0.010	2.37E-16
SLC7A1	high affinity cationic amino acid transporter 1	25648	0.010	2.37E-16
TRAPPC10	trafficking protein particle complex subunit 10	309678	0.010	2.37E-16
EBAG9	PREDICTED: receptor-binding cancer antigen expressed on SiSo cells isoform X1	299864	0.010	2.37E-16
TCF12	transcription factor 12	25720	0.010	2.37E-16
LARP4B	la-related protein 4B	307070	0.010	2.37E-16
SMURF1	PREDICTED: E3 ubiquitin-protein ligase SMURF1 isoform X1	690516	0.010	2.37E-16
CPNE1	copine 1	362249	0.010	2.37E-16
RGD1562394	PREDICTED: 60S ribosomal protein L30-like	364129	0.010	2.37E-16
TMEM126A	PREDICTED: transmembrane protein 126A isoform X1	293113	0.010	2.37E-16

EMC7	PREDICTED: ER membrane protein complex subunit 7 isoform X1	296050	0.010	2.37E-16
SLC25A13	PREDICTED: calcium-binding mitochondrial carrier protein Aralar2 isoform X1	362322	0.010	2.37E-16
RAP1A	ras-related protein Rap-1A precursor	295347	0.010	2.37E-16
COBRA1	negative elongation factor B	311796	0.010	2.37E-16
DHRS7B	dehydrogenase/reductase SDR family member 7B	287380	0.010	2.37E-16
DENND3	PREDICTED: DENN domain-containing protein 3 isoform X1	315055	0.010	2.37E-16
UQCRFS1	cytochrome b-c1 complex subunit Rieske, mitochondrial	291103	0.010	2.37E-16
CBX1	PREDICTED: chromobox protein homolog 1 isoform X1	360609	0.010	2.37E-16
HAT1	PREDICTED: histone acetyltransferase type B catalytic subunit isoform X1	296501	0.010	2.37E-16
SLC25A22	PREDICTED: mitochondrial glutamate carrier 1 isoform X1	309111	0.010	2.37E-16
HP	haptoglobin precursor	24464	0.010	2.37E-16
ANAPC4	anaphase-promoting complex subunit 4	305420	0.010	2.37E-16
WDR12	PREDICTED: ribosome biogenesis protein WDR12 isoform X1	363237	0.010	2.37E-16
LDLRAP1	low density lipoprotein receptor adapter protein 1	500564	0.010	2.37E-16
ATP8A1	PREDICTED: phospholipid-transporting ATPase IA isoform X2	289615	0.010	2.37E-16
DNM1L	PREDICTED: dynamin-1-like protein isoform X1	114114	0.010	2.37E-16
RAB3C	ras-related protein Rab-3C	171058	0.010	2.37E-16
DDX4	probable ATP-dependent RNA helicase DDX4	310090	0.010	2.37E-16
YIF1B	PREDICTED: protein YIF1B isoform X1	103689986	0.010	2.37E-16
KRT19	keratin, type I cytoskeletal 19	360626	0.010	2.37E-16
SSR3	translocon-associated protein subunit gamma	81784	0.010	2.37E-16
MBOAT7	lysophospholipid acyltransferase 7	308309	0.010	2.37E-16
MYL1	PREDICTED: myosin light chain 1/3, skeletal muscle isoform X1	56781	0.010	2.37E-16
TWF2	twinfilin-2	684352	0.010	2.37E-16
LOC679739	PREDICTED: NADH dehydrogenase [ubiquinone] iron-sulfur protein 6, mitochondrial	100912599	0.010	2.37E-16

ABCD1	ATP-binding cassette sub-family D member 1	363516	0.010	2.37E-16
WAPAL	PREDICTED: LOW QUALITY PROTEIN: wings apart-like protein homolog isoform X4	290577	0.010	2.37E-16
KRT2	PREDICTED: keratin, type II cytoskeletal 2 epidermal isoform X1	406228	0.010	2.37E-16
MRPL49	39S ribosomal protein L49, mitochondrial	309176	0.010	2.37E-16
NDUFA7	NADH dehydrogenase [ubiquinone] 1 alpha subcomplex subunit 7	299643	0.010	2.37E-16
EMC6	PREDICTED: ER membrane protein complex subunit 6 isoform X1	287477	0.010	2.37E-16
CES1A	carboxylesterase 1-like precursor	679817	0.010	2.37E-16
TMEM167A	PREDICTED: protein kish-A	100363 808	0.010	2.37E-16
COMMD2	COMM domain-containing protein 2	688478	0.010	2.37E-16
ATP5SL	PREDICTED: ATP synthase subunit s-like protein isoform X1	361520	0.010	2.37E-16
C1QBP	complement component 1 Q subcomponent-binding protein, mitochondrial precursor	29681	0.010	2.37E-16
C2CD2	C2 domain-containing protein 2	304055	0.010	2.37E-16
SLC12A6	solute carrier family 12 member 6	691209	0.010	2.37E-16
ZYG11B	PREDICTED: protein zyg-11 homolog B isoform X1	362559	0.010	2.37E-16
CHCHD4	PREDICTED: mitochondrial intermembrane space import and assembly protein 40	312559	0.010	2.37E-16
KNTC1	PREDICTED: kinetochore-associated protein 1 isoform X1	304477	0.010	2.37E-16
ABCB8	ATP-binding cassette sub-family B member 8, mitochondrial precursor	362302	0.010	2.37E-16
STAMBPL1	AMSH-like protease	687696	0.010	2.37E-16
LOC100359687	PREDICTED: 39S ribosomal protein L1, mitochondrial isoform X1	100359 687	0.010	2.37E-16
MED14	mediator of RNA polymerase II transcription subunit 14	317343	0.010	2.37E-16
NCBP1	nuclear cap-binding protein subunit 1	298075	0.010	2.37E-16
ATP9B	PREDICTED: probable phospholipid-transporting ATPase IIB isoform X1	291411	0.010	2.37E-16
CDC37L1	hsp90 co-chaperone Cdc37-like 1	293886	0.010	2.37E-16
AKAP1	A-kinase anchor protein 1, mitochondrial	114124	0.010	2.37E-16
MFSD5	molybdate-anion transporter precursor	315329	0.010	2.37E-16

MAN1B1	PREDICTED: endoplasmic reticulum mannosyl-oligosaccharide 1,2-alpha-mannosidase isoform X1	499751	0.010	2.37E-16
NDUFA12	NADH dehydrogenase [ubiquinone] 1 alpha subcomplex subunit 12	299739	0.010	2.37E-16
CCDC90B	coiled-coil domain-containing protein 90B, mitochondrial precursor	308820	0.010	2.37E-16
MCU	calcium uniporter protein, mitochondrial precursor	294560	0.010	2.37E-16
MRPL27	39S ribosomal protein L27, mitochondrial	287635	0.010	2.37E-16
CLIC4	chloride intracellular channel protein 4	83718	0.010	2.37E-16
NFATC2IP	NFATC2-interacting protein	308983	0.010	2.37E-16
UQCR10	ubiquinol-cytochrome c reductase complex 7.2kDa protein	685322	0.010	2.37E-16
POLA1	DNA polymerase alpha catalytic subunit	85241	0.010	2.37E-16
TMEM160	transmembrane protein 160 precursor	292654	0.010	2.37E-16
SLC30A9	zinc transporter 9	498358	0.010	2.37E-16
LIMA1	PREDICTED: LIM domain and actin-binding protein 1 isoform X1	300228	0.010	2.37E-16
RGD1307554	uncharacterized protein C19orf47 homolog	292739	0.010	2.37E-16
PRIM1	DNA primase small subunit isoform 1	246327	0.010	2.37E-16
MAPKAPK2	PREDICTED: MAP kinase-activated protein kinase 2 isoform X1	289014	0.010	2.37E-16
ZFR2	PREDICTED: zinc finger RNA-binding protein 2 isoform X1	314639	0.010	2.37E-16
TIMM29	uncharacterized protein C19orf52 homolog	315463	0.010	2.37E-16
CDIPT	PREDICTED: CDP-diacylglycerol--inositol 3-phosphatidyltransferase isoform X1	192260	0.010	2.37E-16

Supplement Table S6: Mitochondrial proteins significantly decreased by 3-day Aβ_{42} treatment (95):

**Protein Symbol**	**Description**	**Log_2 Fold Change**	**p-value**
USMG5	PREDICTED: up-regulated during skeletal muscle growth protein 5	-1.862	5.41E-05
SMURF1	PREDICTED: E3 ubiquitin-protein ligase SMURF1 isoform X1	-6.644	2.37E-16

VDAC3	PREDICTED: voltage-dependent anion-selective channel protein 3 isoform X1	-1.188	2.96E-02
MTCH2	mitochondrial carrier homolog 2 isoform 1x	-1.474	5.34E-06
NDUFV2	NADH dehydrogenase [ubiquinone] flavoprotein 2, mitochondrial precursor	-1.120	4.57E-02
TMEM126A	PREDICTED: transmembrane protein 126A isoform X1	-6.644	2.37E-16
SYNJ2BP	synaptojanin-2-binding protein	-1.419	3.59E-03
SLC25A13	PREDICTED: calcium-binding mitochondrial carrier protein Aralar2 isoform X1	-6.644	2.37E-16
ABCB7	ATP-binding cassette sub-family B member 7, mitochondrial	-1.231	1.72E-02
NDUFC2	NADH dehydrogenase [ubiquinone] 1 subunit C2	-1.336	4.42E-02
VDAC2	PREDICTED: voltage-dependent anion-selective channel protein 2 isoform X1	-0.855	3.40E-02
UQCRC1	cytochrome b-c1 complex subunit 1, mitochondrial precursor	-1.582	3.61E-12
UQCRH	cytochrome b-c1 complex subunit 6, mitochondrial	-2.506	1.41E-12
PHB2	prohibitin-2	-1.591	1.93E-07
COX6C	cytochrome c oxidase subunit 6C-2	-1.751	5.08E-04
NDUFA9	NADH dehydrogenase [ubiquinone] 1 alpha subcomplex subunit 9, mitochondrial	-1.868	5.13E-05
TRIAP1	PREDICTED: TP53-regulated inhibitor of apoptosis 1 isoform X1	-1.498	3.47E-06
NDUFA5	NADH dehydrogenase [ubiquinone] 1 alpha subcomplex subunit 5	-1.020	1.09E-02
NNT	PREDICTED: NAD(P) transhydrogenase, mitochondrial isoform X1	-2.017	4.92E-06
PTPLAD1	very-long-chain (3R)-3-hydroxyacyl-CoA dehydratase 3	-1.117	7.22E-04
ATP5H	ATP synthase subunit d, mitochondrial	-1.932	2.37E-16
COX5A	cytochrome c oxidase subunit 5A, mitochondrial precursor	-0.604	3.20E-02
UQCRB	cytochrome b-c1 complex subunit 7	-1.531	1.14E-05
PDHA1	pyruvate dehydrogenase E1 component subunit alpha, somatic form, mitochondrial precursor	-1.083	1.35E-05
TRIM39	PREDICTED: E3 ubiquitin-protein ligase TRIM39 isoform X1	-0.837	3.54E-02
UQCRFS1	cytochrome b-c1 complex subunit Rieske, mitochondrial	-6.644	2.37E-16
SLC25A1	tricarboxylate transport protein, mitochondrial precursor	-2.152	2.37E-16
ATP5C1	ATP synthase subunit gamma, mitochondrial	-2.458	2.37E-16
APOOL	PREDICTED: MICOS complex subunit MIC27 isoform X1	-1.671	9.74E-07

UQCRQ	cytochrome b-c1 complex subunit 8	-1.791	2.75E-07
FAM162A	PREDICTED: protein FAM162A isoform X1	-1.415	1.42E-10
COX4I2	PREDICTED: cytochrome c oxidase subunit 4 isoform 2, mitochondrial isoform X1	-1.450	7.46E-10
NDUFB10	NADH dehydrogenase [ubiquinone] 1 beta subcomplex subunit 10	-0.908	2.40E-02
SLC25A22	PREDICTED: mitochondrial glutamate carrier 1 isoform X1	-6.644	2.37E-16
AFG3L2	AFG3-like protein 2	-1.065	3.05E-03
SDHC	succinate dehydrogenase cytochrome b560 subunit, mitochondrial	-1.381	8.09E-03
ATP5E	ATP synthase subunit epsilon, mitochondrial	-0.855	4.46E-03
SDHB	succinate dehydrogenase [ubiquinone] iron-sulfur subunit, mitochondrial precursor	-1.470	3.01E-07
TDRKH	PREDICTED: tudor and KH domain-containing protein isoform X1	-0.938	7.93E-03
GHR	PREDICTED: growth hormone receptor isoform X1	-1.470	1.18E-02
SDHA	succinate dehydrogenase [ubiquinone] flavoprotein subunit, mitochondrial precursor	-1.333	2.05E-09
ATP5J2	ATP synthase subunit f, mitochondrial	-2.077	2.37E-16
NDUFA10	NADH dehydrogenase [ubiquinone] 1 alpha subcomplex subunit 10, mitochondrial precursor	-1.023	1.05E-02
LETM1	LETM1 and EF-hand domain-containing protein 1, mitochondrial precursor	-0.980	7.45E-04
VDAC1	voltage-dependent anion-selective channel protein 1	-1.431	1.80E-09
ATP5B	ATP synthase subunit beta, mitochondrial precursor	-2.041	2.37E-16
TOMM70A	mitochondrial import receptor subunit TOM70	-1.155	4.46E-05
MRPL9	39S ribosomal protein L9, mitochondrial	-1.515	3.44E-02
PDHB	pyruvate dehydrogenase E1 component subunit beta, mitochondrial precursor	-1.117	5.06E-06
NDUFA13	NADH dehydrogenase [ubiquinone] 1 alpha subcomplex subunit 13	-3.506	2.37E-16
ATP5D	PREDICTED: ATP synthase subunit delta, mitochondrial isoform X1	-3.047	2.37E-16
EMC2	ER membrane protein complex subunit 2	-1.158	2.10E-03
COMTD1	catechol O-methyltransferase domain-containing protein 1	-1.290	2.39E-03
CISD1	CDGSH iron-sulfur domain-containing protein 1	-1.502	7.74E-07
ATP5F1	ATP synthase F(0) complex subunit B1, mitochondrial precursor	-2.315	2.37E-16
FIS1	PREDICTED: mitochondrial fission 1 protein isoform X1	-1.105	2.99E-03
TAP1	antigen peptide transporter 1 precursor	-1.404	1.35E-04

DLAT	dihydrolipoyllysine-residue acetyltransferase component of pyruvate dehydrogenase complex, mitochondrial	-1.184	1.17E-05
SLC25A4	ADP/ATP translocase 1	-2.826	2.37E-16
ATP5L	ATP synthase subunit g, mitochondrial	-2.146	2.37E-16
PHB	prohibitin	-1.966	2.37E-16
UQCRC2	cytochrome b-c1 complex subunit 2, mitochondrial precursor	-1.340	4.94E-06
ATP5O	ATP synthase subunit O, mitochondrial precursor	-2.065	2.37E-16
SLC25A11	mitochondrial 2-oxoglutarate/malate carrier protein	-2.300	5.25E-13
SLC25A5	ADP/ATP translocase 2	-2.245	2.37E-16
SLC25A10	mitochondrial dicarboxylate carrier	-1.191	8.09E-06
ATP5I	ATP synthase subunit e, mitochondrial	-1.737	8.81E-12
MPC2	mitochondrial pyruvate carrier 2 isoform 1	-1.751	3.03E-08
ATP5A1	ATP synthase subunit alpha, mitochondrial precursor	-1.857	2.37E-16
SLC25A12	PREDICTED: LOW QUALITY PROTEIN: calcium-binding mitochondrial carrier protein Aralar1 isoform X2	-1.791	7.87E-13
SLC25A3	phosphate carrier protein, mitochondrial isoform 2 precursor	-2.531	2.37E-16
CYC1	cytochrome c-1	-2.095	3.98E-12
SLC25A24	calcium-binding mitochondrial carrier protein SCaMC-1	-1.723	1.98E-11
MRPL17	39S ribosomal protein L17, mitochondrial isoform 1 precursor	-1.635	1.22E-03
SORD	sorbitol dehydrogenase	-1.077	4.69E-02
DNM1L	PREDICTED: dynamin-1-like protein isoform X1	-6.644	2.37E-16
KRAS	PREDICTED: GTPase KRas isoform X3	-1.544	4.83E-02
LOC679739	PREDICTED: NADH dehydrogenase [ubiquinone] iron-sulfur protein 6, mitochondrial	-6.644	2.37E-16
ABCD1	ATP-binding cassette sub-family D member 1	-6.644	2.37E-16
MRPL49	39S ribosomal protein L49, mitochondrial	-6.644	2.37E-16
NDUFA7	NADH dehydrogenase [ubiquinone] 1 alpha subcomplex subunit 7	-6.644	2.37E-16
ATP5SL	PREDICTED: ATP synthase subunit s-like protein isoform X1	-6.644	2.37E-16
C1QBP	complement component 1 Q subcomponent-binding protein, mitochondrial precursor	-6.644	2.37E-16
CHCHD4	PREDICTED: mitochondrial intermembrane space import and assembly protein 40	-6.644	2.37E-16
ABCB8	ATP-binding cassette sub-family B member 8, mitochondrial precursor	-6.644	2.37E-16
LOC100359687	PREDICTED: 39S ribosomal protein L1, mitochondrial isoform X1	-6.644	2.37E-16

NCBP1	nuclear cap-binding protein subunit 1	-6.644	2.37E-16
AKAP1	A-kinase anchor protein 1, mitochondrial	-6.644	2.37E-16
NDUFA12	NADH dehydrogenase [ubiquinone] 1 alpha subcomplex subunit 12	-6.644	2.37E-16
CCDC90B	coiled-coil domain-containing protein 90B, mitochondrial precursor	-6.644	2.37E-16
MCU	calcium uniporter protein, mitochondrial precursor	-6.644	2.37E-16
MRPL27	39S ribosomal protein L27, mitochondrial	-6.644	2.37E-16
CLIC4	chloride intracellular channel protein 4	-6.644	2.37E-16
UQCR10	ubiquinol-cytochrome c reductase complex 7.2kDa protein	-6.644	2.37E-16
TMEM160	transmembrane protein 160 precursor	-6.644	2.37E-16

Supplement Table S7: Mitochondrial proteins significantly increased by 3-day Aβ_{42} treatment (13)

**Protein Symbol**	**Description**	**Log_2 Fold Change**	**p-value**
CMC1	PREDICTED: COX assembly mitochondrial protein homolog isoform X1	2.71	8.19E-03
PIGY	protein preY, mitochondrial precursor	3.54	1.26E-04
ATP5J	PREDICTED: ATP synthase-coupling factor 6, mitochondrial isoform X1	2.88	2.39E-03
TIMM8B	mitochondrial import inner membrane translocase subunit Tim8 B	2.16	7.44E-03
MYH9	myosin-9	2.10	1.40E-05
PSAP	prosaposin isoform B preproprotein	1.74	3.48E-03
GSTP1	glutathione S-transferase P	1.85	1.47E-03
DBI	acyl-CoA-binding protein	3.21	8.40E-14
CROT	PREDICTED: peroxisomal carnitine O-octanoyltransferase isoform X1	3.37	3.36E-04
PEBP1	phosphatidylethanolamine-binding protein 1	1.75	2.98E-03
BLOC1S2	PREDICTED: biogenesis of lysosome-related organelles complex-1 subunit 2 isoform X2	3.48	4.28E-04
GTF3C4	general transcription factor 3C polypeptide 4	100.00	2.37E-16
PDP1	PREDICTED: pyruvate dehydrogenase [acetyl-transferring]-phosphatase 1, mitochondrial isoform X1	100.00	2.37E-16

Supplement Table S8**:** Nuclear proteins significantly decreased by 3-day Aβ_{42} treatment (64)

Protein Symbol	Description	Log_2 Fold Change	p-value
LBR	lamin-B receptor	-1.272	2.68E-02
CBX3	PREDICTED: chromobox protein homolog 3 isoform X1	-6.644	2.37E-16
TCF12	transcription factor 12	-6.644	2.37E-16
WARS	PREDICTED: tryptophan--tRNA ligase, cytoplasmic isoform X1	-1.221	4.51E-02
RBM3	PREDICTED: RNA-binding protein 3 isoform X2	-1.523	4.88E-02
VDAC3	PREDICTED: voltage-dependent anion-selective channel protein 3 isoform X1	-1.188	2.96E-02
MTCH2	mitochondrial carrier homolog 2 isoform 1x	-1.474	5.34E-06
CPNE1	copine 1	-6.644	2.37E-16
RFC5	replication factor C subunit 5	-1.462	4.96E-03
PPARD	PREDICTED: peroxisome proliferator-activated receptor delta isoform X1	-2.000	8.57E-06
NOSIP	nitric oxide synthase-interacting protein	-1.358	2.77E-02
VDAC2	PREDICTED: voltage-dependent anion-selective channel protein 2 isoform X1	-0.855	3.40E-02
COBRA1	negative elongation factor B	-6.644	2.37E-16
PHB2	prohibitin-2	-1.591	1.93E-07
NDUFA9	NADH dehydrogenase [ubiquinone] 1 alpha subcomplex subunit 9, mitochondrial	-1.868	5.13E-05
TOR1AIP1	torsin-1A-interacting protein 1	-1.248	2.05E-02
ZFP819	PREDICTED: zinc finger protein 175 isoform X2	-1.214	3.82E-05
PDHA1	pyruvate dehydrogenase E1 component subunit alpha, somatic form, mitochondrial precursor	-1.083	1.35E-05
SLC25A1	tricarboxylate transport protein, mitochondrial precursor	-2.152	2.37E-16
CBX1	PREDICTED: chromobox protein homolog 1 isoform X1	-6.644	2.37E-16
TMPO	lamina-associated polypeptide 2, isoform beta	-1.077	5.78E-03
LMNA	prelamin-A/C	-1.155	6.81E-07
GOLGA2	PREDICTED: golgin subfamily A member 2 isoform X1	-0.852	4.55E-02
TMEM43	transmembrane protein 43	-1.208	1.35E-03
TAGLN3	transgelin-3	-0.966	1.26E-04
FUBP3	PREDICTED: far upstream element-binding protein 3 isoform X1	-0.905	2.17E-02
GHR	PREDICTED: growth hormone receptor isoform X1	-1.470	1.18E-02
ANAPC4	anaphase-promoting complex subunit 4	-6.644	2.37E-16
ATP5J2	ATP synthase subunit f, mitochondrial	-2.077	2.37E-16
CFL1	cofilin-1	-0.597	3.54E-02
VDAC1	voltage-dependent anion-selective channel protein 1	-1.431	1.80E-09

ATP5B	ATP synthase subunit beta, mitochondrial precursor	-2.041	2.37E-16
PDHB	pyruvate dehydrogenase E1 component subunit beta, mitochondrial precursor	-1.117	5.06E-06
NDUFA13	NADH dehydrogenase [ubiquinone] 1 alpha subcomplex subunit 13	-3.506	2.37E-16
EMC2	ER membrane protein complex subunit 2	-1.158	2.10E-03
ATP5F1	ATP synthase F(0) complex subunit B1, mitochondrial precursor	-2.315	2.37E-16
TMEM120A	transmembrane protein 120A	-1.127	1.28E-03
WDR12	PREDICTED: ribosome biogenesis protein WDR12 isoform X1	-6.644	2.37E-16
PAFAH1B1	PREDICTED: platelet-activating factor acetylhydrolase IB subunit alpha isoform X1	-0.573	4.88E-02
SLC25A4	ADP/ATP translocase 1	-2.826	2.37E-16
ALB	serum albumin precursor	-1.431	7.83E-11
PHB	prohibitin	-1.966	2.37E-16
ATP5O	ATP synthase subunit O, mitochondrial precursor	-2.065	2.37E-16
SLC25A11	mitochondrial 2-oxoglutarate/malate carrier protein	-2.300	5.25E-13
SLC25A5	ADP/ATP translocase 2	-2.245	2.37E-16
SLC25A10	mitochondrial dicarboxylate carrier	-1.191	8.09E-06
MPC2	mitochondrial pyruvate carrier 2 isoform 1	-1.751	3.03E-08
ACTG1	PREDICTED: actin, cytoplasmic 2 isoform X1	-0.648	1.71E-02
RTRAF	PREDICTED: UPF0568 protein C14orf166 homolog isoform X1	-0.628	4.38E-02
CYC1	cytochrome c-1	-2.095	3.98E-12
DDX4	probable ATP-dependent RNA helicase DDX4	-6.644	2.37E-16
WAPAL	PREDICTED: LOW QUALITY PROTEIN: wings apart-like protein homolog isoform X4	-6.644	2.37E-16
KRT2	PREDICTED: keratin, type II cytoskeletal 2 epidermal isoform X1	-6.644	2.37E-16
C1QBP	complement component 1 Q subcomponent-binding protein, mitochondrial precursor	-6.644	2.37E-16
C2CD2	C2 domain-containing protein 2	-6.644	2.37E-16
KRT10	PREDICTED: keratin, type I cytoskeletal 10 isoform X1	-1.786	1.47E-03
ABCB8	ATP-binding cassette sub-family B member 8, mitochondrial precursor	-6.644	2.37E-16
MED14	mediator of RNA polymerase II transcription subunit 14	-6.644	2.37E-16
NCBP1	nuclear cap-binding protein subunit 1	-6.644	2.37E-16
CLIC4	chloride intracellular channel protein 4	-6.644	2.37E-16
NFATC2IP	NFATC2-interacting protein	-6.644	2.37E-16
POLA1	DNA polymerase alpha catalytic subunit	-6.644	2.37E-16
SLC30A9	zinc transporter 9	-6.644	2.37E-16

MAPKAPK2	PREDICTED: MAP kinase-activated protein kinase 2 isoform X1	-6.644	2.37E-16

Supplement Table S9: Nuclear proteins significantly increased by 3-day $A\beta_{42}$ treatment (22)

**Protein Symbol**	**Description**	**Log_2 Fold Change**	**p-value**
ATR	PREDICTED: serine/threonine-protein kinase ATR isoform X3	0.986	1.01E-04
PKIA	PREDICTED: cAMP-dependent protein kinase inhibitor alpha isoform X1	1.496	2.36E-01
JPT1	hematological and neurological expressed 1 protein	1.938	3.29E-10
PDXK	pyridoxal kinase	1.416	3.32E-01
FAT1	PREDICTED: protocadherin Fat 1 isoform X1	0.749	2.55E-02
UFM1	ubiquitin-fold modifier 1 precursor	1.530	8.23E-05
NUTF2	nuclear transport factor 2	0.723	1.15E-01
UBQLN1	ubiquilin-1	0.677	1.47E-01
HINT1	histidine triad nucleotide-binding protein 1	1.732	7.77E-10
MYH9	myosin-9	1.069	9.46E-05
MTPN	myotrophin	1.062	2.87E-02
GSTP1	glutathione S-transferase P	0.888	1.08E-01
SCG5	neuroendocrine protein 7B2 precursor	1.259	5.87E-03
DBI	acyl-CoA-binding protein	1.681	2.40E-09
PPIA	PREDICTED: peptidyl-prolyl cis-trans isomerase A	0.662	1.04E-01
DPY30	protein dpy-30 homolog	1.365	3.27E-05
PEBP1	phosphatidylethanolamine-binding protein 1	0.811	6.71E-02
RPS27A	ubiquitin-40S ribosomal protein S27a	1.141	4.65E-06
BLOC1S2	PREDICTED: biogenesis of lysosome-related organelles complex-1 subunit 2 isoform X2	1.799	7.68E-01
TSC22D1	TSC22 domain family protein 1 isoform 1	6.644	2.12E-16
GTF3C4	general transcription factor 3C polypeptide 4	100.00	2.12E-16
NSRP1	nuclear speckle splicing regulatory protein 1	100.00	2.12E-16

Supplement Table S10: Genomic Enrichment Analysis Results

<table>
<tr><th>Chromosome Region</th><th>Percent Overlap</th><th>Overlapping Entities</th><th>p-value</th><th>Associated Phenotype</th><th>Number Genes on the Chromosome</th></tr>
<tr><td>1p13.2</td><td>1</td><td>ATP5PB
RAP1A</td><td>2.33E-02</td><td>Autism spectrum disorder</td><td rowspan="3">18</td></tr>
<tr><td>1p13.3</td><td>1</td><td>SLC25A24
RAP1A</td><td>2.66E-02</td><td>Coronary artery disease</td></tr>
<tr><td>1p33-p32</td><td>22</td><td>AKR1A1</td><td>1.76E-04</td><td>Renal hypodysplasia</td></tr>
</table>

		EPB41			
1p34.1	1	*AKR1A1*<br>*HPDL*	2.16E-02	Hypercholesterolemia	
1p36.1	10	*DNAJC16*<br>*DDOST*	8.26E-04	Developmental and intellectual delay and deficits<br>Seizures<br>Cranio-facial abnormalities	
1p36.11	1	*CLIC4*<br>*LDLRAP1*	3.99E-02		
1p36.13	1	*EMC1*<br>*SDHB*	3.35E-02		
1p36.21	1	*DNAJC16*<br>*UQCRHL*	2.16E-02		
1q21	3	*MRPL9*<br>*PGLYRP3*<br>*TDRKH*	1.05E-03	Developmental delay<br>Epilepsy<br>Microcephaly	
1q21.3	1	*PGLYRP3*<br>*TDRKH*<br>*MRPL9*	1.67E-02		
1q24.2	2	*TOR1AIP1*<br>*MPC2*	1.30E-02	Microcephaly<br>Intellectual deficit<br>Short stature	
2q37.3	1	*SCLY*<br>*NDUFA10*	4.41E-02	Brachydactyly<br>Schizophrenia<br>Round face	2
3p25.1	4	*TMEM43*<br>*CHCHD4*<br>*C3ORF20*	4.94E-04	Breast cancer recurrence	7
3q21.1	6	*PARP14*<br>*FAM162A*	2.07E-03	Type 2 Diabetes<br>Abnormal bone mineral density	
3q25.1	2	*PFN2*<br>*COMMD2*	1.30E-02	Microcephaly<br>Developmental delay<br>Facial dysmorphism	
4p13	4	*SLC30A9*<br>*ATP8A1*	4.21E-03	Autism spectrum disorder<br>Developmental delays	4
4q35	15	*FAT1*<br>*SLC25A4*	3.80E-04	Facio-Scapulo-Humeral Dystrophy	
5p15.33	2	*NDUFS6*<br>*SDHA*	1.71E-02	Cri-Du-Chat Syndrome<br>Lung cancer	8
5q11.2	1	*RAB3C*<br>*DDX4*	2.93E-02	Developmental delay<br>Heart defects<br>Short stature	
5q31.1	1	*VDAC1*<br>*UQCRQ*	3.64E-02	Developmental delay<br>Short stature<br>Encephalopathy	

				Congenital heart defects	
5q32	2	*ATOX1*<br>*DPYSL3*	2.08E-02	Schizophrenia<br>Netherton syndrome	
6p21.3	1	*TRIM39*<br>*TAP1*<br>*CCHCR1*	1.48E-02	Autoimmune disease	3
7q22.1	1	*SMURF1*<br>*ATP5MF*<br>*FIS1*	1.25E-02	Myelodysplasia<br>Acute myeloid leukemia	5
7q31.32	5	*CADPS2*<br>*NDUFA5*	3.13E-03	Autism spectrum disorder<br>Psychosis	
9p24.1	2	*KIAA2026*<br>*CDC37L1*	1.23E-02	Hodgkin lymphoma<br>Schizoaffective disorder<br>Bipolar disorder	6
9q34	3	*NELFB*<br>*MAN1B1*	6.33E-03	Intellectual deficits<br>Epilepsy	
9q34.11	1	*FUBP3*<br>*GOLGA2*	3.02E-02		
10q22.1	1	*MCU*<br>*PSAP*	2.12E-02	Autism spectrum disorder	6
10q22.2	2	*VDAC2*<br>*COMTD1*	1.08E-02		
10q24.1	3	*TM9SF3*<br>*SFRP5*	6.10E-03	Ectrodactyly	
11q13	2	*MRPL49*<br>*GSTP1*<br>*CFL1*	1.88E-03	Breast cancer<br>Oropharyngeal carcinoma	12
11q13.1	1	*MRPL49*<br>*CFL1*<br>*PPP2R5B*	5.77E-03		
11q14.1	3	*TMEM126A*<br>*NDUFC2*<br>*CCDC90B*	1.47E-03	Microcephaly<br>Developmental delay<br>Short stature	
11q22.3	1	*DCUN1D5*<br>*MMP10*	2.20E-02	Intellectual deficits<br>Developmental delay<br>Facial dysmorphism	
11q23.1	4	*ALG9*<br>*DLAT*<br>*TIMM8B*	5.37E-04	Breast cancer	
12p13.3	7	*ERC1*<br>*NDUFA9*	1.80E-03	Hypertension<br>Nasopharyngeal carcinoma	13

12q13.3	2	*NDUFA4L2*<br>*ATP5F1B*	1.57E-02	Craniofacial abnormalities<br>Sarcoidosis	
12q13.12	2	*TROAP*<br>*LIMA1*	1.97E-02	Asthma	
12q22	2	*NDUFA12*<br>*TMPO*	1.46E-02	Major depressive disorder	
12q23.1	2	*SLC25A3*<br>*TMPO*	1.14E-02	Major depressive disorder<br>Darier's disease	
12q24.23	3	*RFC5*<br>*PEBP1*	6.33E-03	Facial dysmorphism<br>Developmental delay<br>Heart defects	
12q24.31	1	*KNTC1*<br>*MLEC*	4.78E-02	Developmental delay<br>Facial dysmorphisms	
13q32.3	3	*TM9SF2*<br>*GGACT*	7.04E-03	Craniofacial dysmorphisms<br>Intellectual deficits<br>Cardiac defects<br>Holoprosencephaly	2
14q24.3	1	*NPC2*<br>*TMED10*	4.46E-02	Facial dysmorphism<br>Developmental delay<br>Epilepsy	2
15q14	2	*SLC12A6*<br>*ACTC1*<br>*EMC7*	1.69E-03	Autism spectrum disorder<br>Heart defect<br>Developmental delay	5
15q21.1	2	*SORD*<br>*BLOC1S6*	1.89E-02	Breast cancer	
16q22.2	4	*HP*<br>*PHLPP2*	5.22E-03	Facial dysmorphism<br>Intellectual deficit<br>Schizophrenia<br>Epilepsy	2
17p13.2	2	*SLC25A11*<br>*EMC6*<br>*C1QBP*	2.20E-03	Cholangiocarcinoma	11
17p13.3	3	*SLC43A2*<br>*SLC25A11*<br>*PAFAH1B1*<br>*C1QBP*	2.27E-04	Autism spectrum disorder<br>Intellectual deficits<br>Craniofacial abnormalities	
17q21	3	*PHB*<br>*ATP6V0A1*	6.33E-03	Breast cancer<br>Developmental delay<br>Kyphoscoliosis	
17q21.33	2	*PHB*<br>*MRPL27*	1.20E-02		
17q25	5	*ATP5H*	3.30E-03	Esophageal cancer	

		ACTG1		Pancreatic cancer	
17q25.1	1	*ATP5H*<br>*HN1*	4.46E-02		
18p11.22	3	*NDUFV2*<br>*VAPA*	6.10E-03	Lung cancer	6
18p11.31	2	*EPB41L3*<br>*MYL12B*	1.02E-02	Microcephaly<br>Intellectual deficits<br>Craniofacial abnormalities	
18q21.1	2	*ATP5F1A*<br>*HDHD2*	1.78E-02	Pancreatic Cancer	
19p13.11	1	*ATP13A1*<br>*NDUFA13*	4.57E-02	Ectrodactyly	11
19p13.2	1	*SLC44A2*<br>*NDUFA7*<br>*TIMM29*<br>*NDUFA13*	5.99E-03	Developmental delay<br>Craniofacial dysmorphisms	
19p13.3	0	*MYDGF*<br>*ZFR2*<br>*ATP5F1D*	3.74E-02		
19q13.33	1	*MYH14*<br>*NOSIP*<br>*NAPA*	1.48E-02	Developmental delay<br>Intellectual deficits	
20q11.21	1	*TM9SF4*<br>*COX4I2*	2.20E-02	Intellectual deficits<br>Facial dysmorphisms	4
20q11.22	2	*ACSS2*<br>*CPNE1*	1.23E-02	Developmental delay<br>Intellectual deficits<br>Facial dysmorphisms<br>Melanoma	
21q22.3	1	*PDXK*<br>*C2CD2*<br>*TRAPPC10*	1.14E-02	Down's syndrome<br>Autism spectrum disorder<br>Facial dysmorphisms	3
22q12.2	1	*UQCR10*<br>*PLA2G3*	2.20E-02	Craniofacial dysmorphism<br>Microcephaly<br>Developmental delay<br>Intellectual deficits	2
Xp11.23	1	*RBM3*<br>*PLP2*	4.20E-02	Intellectual deficit<br>Developmental delay<br>Syndactyly	2

Supplement Table S11: ER proteins significantly decreased by 3-day Aβ_{42} treatment (33):

Protein Symbol	Description	Log_2 Fold Change	p-value
SSR1	PREDICTED: translocon-associated protein subunit alpha isoform X1	-1.258	2.17E-04
ALG9	PREDICTED: alpha-1,2-mannosyltransferase ALG9 isoform X1	-1.234	3.51E-02
VDAC3	PREDICTED: voltage-dependent anion-selective channel protein 3 isoform X1	-1.188	2.96E-02
VAPA	PREDICTED: vesicle-associated membrane protein-associated protein A isoform X1	-0.727	3.79E-02
OSTC	oligosaccharyltransferase complex subunit OSTC	-1.279	5.78E-03
DHRS7B	dehydrogenase/reductase SDR family member 7B	-6.644	2.37E-16
PTPLAD1	very-long-chain (3R)-3-hydroxyacyl-CoA dehydratase 3	-1.117	7.22E-04
MEST	mesoderm-specific transcript homolog protein	-0.870	4.08E-02
SSR4	translocon-associated protein subunit delta precursor	-0.847	8.35E-03
DDOST	dolichyl-diphosphooligosaccharide--protein glycosyltransferase 48 kDa subunit precursor	-0.793	1.44E-03
TMED10	transmembrane emp24 domain-containing protein 10 precursor	-0.651	1.65E-02
TMEM43	transmembrane protein 43	-1.208	1.35E-03
HP	haptoglobin precursor	-6.644	2.37E-16
TAP2	antigen peptide transporter 2 precursor	-0.977	1.08E-02
EMC2	ER membrane protein complex subunit 2	-1.158	2.10E-03
FIS1	PREDICTED: mitochondrial fission 1 protein isoform X1	-1.105	2.99E-03
TAP1	antigen peptide transporter 1 precursor	-1.404	1.35E-04
RPN1	dolichyl-diphosphooligosaccharide--protein glycosyltransferase subunit 1 precursor	-0.651	1.64E-02
CKAP4	PREDICTED: cytoskeleton-associated protein 4 isoform X1	-0.813	9.58E-04
ALB	serum albumin precursor	-1.431	7.83E-11
RPN2	PREDICTED: dolichyl-diphosphooligosaccharide--protein glycosyltransferase subunit 2 isoform X1	-1.370	6.20E-10
TMED7	transmembrane emp24 domain-containing protein 7 precursor	-1.482	4.16E-08
RAB1	ras-related protein Rab-1A	-0.630	2.19E-02
EMC1	PREDICTED: ER membrane protein complex subunit 1 isoform X1	-0.881	3.27E-03
MLEC	malectin precursor	-1.211	1.57E-03
ATP8A1	PREDICTED: phospholipid-transporting ATPase IA isoform X2	-6.644	2.37E-16
DNM1L	PREDICTED: dynamin-1-like protein isoform X1	-6.644	2.37E-16
SSR3	translocon-associated protein subunit gamma	-6.644	2.37E-16

EMC6	PREDICTED: ER membrane protein complex subunit 6 isoform X1	-6.644	2.37E-16
MAN1B1	PREDICTED: endoplasmic reticulum mannosyl-oligosaccharide 1,2-alpha-mannosidase isoform X1	-6.644	2.37E-16
CLIC4	chloride intracellular channel protein 4	-6.644	2.37E-16
SLC30A9	zinc transporter 9	-6.644	2.37E-16
CDIPT	PREDICTED: CDP-diacylglycerol--inositol 3-phosphatidyltransferase isoform X1	-6.644	2.37E-16

Supplement Table S12: ER proteins significantly decreased by 3-day Aβ_{42} treatment (9)

**Protein Symbol**	**Description**	**Log_2 Fold Change**	**p-value**
VKORC1L 1	PREDICTED: vitamin K epoxide reductase complex subunit 1-like protein 1 isoform X1	1.207	4.36E-03
UFM1	ubiquitin-fold modifier 1 precursor	1.530	2.93E-06
UBQLN1	ubiquilin-1	0.677	4.57E-02
ERC1	PREDICTED: ELKS/Rab6-interacting/CAST family member 1 isoform X1	2.352	1.22E-11
HHATL	protein-cysteine *N*-palmitoyltransferase HHAT-like protein	2.144	1.32E-11
FKBP1A	peptidyl-prolyl cis-trans isomerase FKBP1A	2.212	2.37E-16
CYP51	lanosterol 14-alpha demethylase	0.631	4.78E-02
DBI	acyl-CoA-binding protein	1.681	8.40E-14
PEBP1	phosphatidylethanolamine-binding protein 1	0.811	2.98E-03
